# Interaction of helminth parasites with the haemostatic system of their vertebrate hosts: a scoping review[Fn FN1]

**DOI:** 10.1051/parasite/2022034

**Published:** 2022-07-14

**Authors:** Alicia Diosdado, Fernando Simón, Judit Serrat, Javier González-Miguel

**Affiliations:** 1 Laboratory of Parasitology, Faculty of Pharmacy, University of Salamanca 37007 Salamanca Spain; 2 Laboratory of Parasitology, Institute of Natural Resources and Agrobiology of Salamanca (IRNASA-CSIC) 37008 Salamanca Spain; 3 Molecular Parasitology Laboratory, Centre of One Health (COH), Ryan Institute, National University of Ireland H91 DK59 Galway Ireland

**Keywords:** Helminth parasites, Haemostatic system, Coagulation, Fibrinolysis, Host–parasite relationships, Scoping review

## Abstract

Helminth parasitoses are among the most prevalent health issues worldwide. Their control depends largely on unravelling host–parasite interactions, including parasitic exploitation of the host haemostatic system. The present study undertakes a scoping review of the research carried out in this field with the aim of unifying and updating concepts. Multiple keywords combined with Boolean operators were employed to design the literature search strategy. Two online databases were used to identify original peer-reviewed articles written in English and published before 1st January 2020 describing molecular interactions between helminth parasites and the host haemostatic system. Relevant data from the selected sources of evidence were extracted and analysed. Ninety-six publications reporting 259 interactions were selected. Fifty-three proteins belonging to 32 species of helminth parasites were involved in interactions with components of the host haemostatic system. Many of these proteins from both parasite and host were conserved among the different interactions identified. Most of these interactions were related to the inhibition of the coagulation system and the activation of fibrinolysis. This was associated mainly with a potential of parasites to reduce the formation of blood clots in the host and attributed to biological processes, such as parasite nutrition, survival, invasion, evasion and migration or the appearance of pathological mechanisms in the host. A wide range of helminth parasites have developed similar strategies to exploit the haemostatic system of their hosts, which could be regarded as an evolutionary conserved mechanism that could confer benefits to parasites in terms of survival and establishment in their vertebrate hosts.

## Abstract (fr)

Les parasitoses par les helminthes sont à l’origine de problèmes de santé parmi les plus répandus dans le monde. Leur contrôle dépend en grande partie du démêlage des interactions hôte-parasite, y compris l’exploitation par les parasites du système hémostatique de l’hôte. La présente étude entreprend un examen exploratoire des recherches menées dans ce domaine dans le but d’unifier et d’actualiser les concepts. Plusieurs mots-clés combinés à des opérateurs booléens ont été utilisés pour concevoir la stratégie de recherche documentaire. Deux bases de données en ligne ont été utilisées pour identifier des articles originaux évalués par des pairs rédigés en anglais et publiés avant le 1er janvier 2020, décrivant les interactions moléculaires entre les helminthes parasites et le système hémostatique de l’hôte. Les données pertinentes des sources sélectionnées ont été extraites et analysées. Quatre-vingt-seize publications rapportant 259 interactions ont été sélectionnées. Cinquante-trois protéines appartenant à 32 espèces d’helminthes parasites ont été impliquées dans des interactions avec des composants du système hémostatique de l’hôte. Beaucoup de ces protéines du parasite et de l’hôte ont été conservées parmi les différentes interactions identifiées. La plupart de ces interactions étaient liées à l’inhibition du système de coagulation et à l’activation de la fibrinolyse. Ceci était principalement associé à un potentiel des parasites à réduire la formation de caillots sanguins chez l’hôte et attribué à des processus biologiques, tels que la nutrition, la survie, l’invasion, l’évasion et la migration des parasites ou l’apparition de mécanismes pathologiques chez l’hôte. Un large éventail d’helminthes parasites ont développé des stratégies similaires pour exploiter le système hémostatique de leurs hôtes, ce qui pourrait être considéré comme un mécanisme évolutif conservé qui pourrait conférer des avantages aux parasites en termes de survie et d’établissement chez leurs hôtes vertébrés.

## Introduction

Parasites evolved from free-living ancestors millions of years ago; therefore, they have developed adaptations to their parasitic life styles. In terms of evolutionary convergence, some key mechanisms have been perpetuated and similarities between distantly related groups in which parasitism had independent origins are commonly reported. An example of these adaptations is the sophisticated strategies that different groups of parasites have developed in order to facilitate host exploitation and the control of host physiology for their own benefit [46]. In this context, the ability of parasites to utilise the host haemostatic system has been documented since the beginning of the 20th century [34].

The haemostatic system comprises the mechanisms responsible for maintaining the normal functioning of blood vessels in vertebrates by restoring the integrity of the vascular wall when it is disrupted. These mechanisms are classified into two interrelated systems depending on whether they are directed at the formation of a blood clot to seal the defect (coagulation) or at its dissolution to restore the normal vascular state (fibrinolysis). When a blood vessel injury occurs, flowing blood comes into contact with vascular wall structures that are different from the endothelium (sub-endothelial matrix and collagen), triggering the adhesion of platelets to the injury site, a process in which the von Willebrand factor (vWF) plays a pivotal role. Adhering platelets are activated and form an aggregate on which a network of fibrin is deposited, resulting in the formation of a blood clot and healing of the injured vascular wall [4]. Fibrin is the final product of the coagulation cascade, a series of enzymatic chain reactions in which different zymogens (coagulation factors) are activated into their active serine proteases by action of previously activated coagulation factors. The coagulation cascade consists of two interconnected pathways: the extrinsic pathway, initiated when sub-endothelial tissue factor (TF) is expressed as a result of endothelial damage or endothelial activation by chemicals or inflammatory processes, and the intrinsic pathway, which begins with the activation of factor XII (FXIIa) when blood comes into contact with a surface different from the endothelium. Both the extrinsic and intrinsic pathways converge into a common pathway with the activation of factor X (FXa), a key factor for thrombin formation. Thrombin is the enzyme that finally transforms soluble fibrinogen into insoluble fibrin, which is cross-linked by the activated factor XIII (FXIIIa). Blood coagulation is attenuated by several inhibitors among which antithrombin III (AT-III) is the most quantitatively important by neutralizing all serine proteases produced during the coagulation process. Other inhibitors of this system are the tissue factor pathway inhibitor (TFPI) and the activated protein C (APC) [1, 9, 52]. Upon healing of the injured blood vessel, the clot is dissolved through conversion of the insoluble fibrin network to soluble fibrin degradation products by the action of plasmin. Plasmin is the catalytically active enzyme of plasminogen, the central proenzyme of the fibrinolytic system. The conversion of plasminogen into plasmin occurs by tissue-type (tPA) and urokinase-type (uPA) plasminogen activators after plasminogen binding to fibrin. This process is inhibited by plasminogen activator inhibitors 1 (PAI-1) and 2 (PAI-2) at the level of tPA and uPA and by α2-antiplasmin (A2AP) at the level of plasmin [10, 11, 33].

So far, many original articles and several narrative reviews [5, 16, 22, 30, 39, 41, 61] have highlighted the interaction of helminth parasites with the coagulation or fibrinolytic systems of their hosts. Nonetheless, to our knowledge, no review collecting all available information in the field has been published. The present work aimed to carry out a scoping review on the molecular interaction between helminth parasites and the haemostatic system of their vertebrate hosts in order to systematically and homogeneously review, unify and summarize the evidence published in the field, update concepts, identify knowledge gaps and contribute to future research.

## Materials and methods

### Protocol and eligibility criteria

The present scoping review was conducted following a protocol based on the Preferred Reporting Items for Systematic reviews and Meta-Analyses extension for Scoping Reviews (PRISMA-ScR) guidelines [56] (Supplementary Checklist). To be included in the review, peer-reviewed journal papers needed to be published prior to 1st January 2020. After establishing this condition, the eligibility of sources of evidence was based on seven additional criteria: (i) publications needed to be written in English; (ii) papers were required to be published as original articles (reviews, meta-analysis, books, clinical trials, case reports, conference papers, editorials, comments, letters or guidelines were excluded from the study); (iii) the titles and abstracts of the articles needed to fit the scope of the review (they were required to study interactions between helminth parasites and the haemostatic system of their vertebrate hosts); (iv) papers needed to have a full text available; (v) studies were required to assess molecular interactions through *in vitro*/*ex vivo* experiments (articles that only published *in vivo* test results were excluded from the study); (vi) papers needed to study interactions between helminth parasites and the main components of the haemostatic system of vertebrates (reviewed in [4]); and (vii) articles were required to empirically demonstrate a host–parasite interaction (publications that studied/suggested interactions without experimental basis were excluded from the analysis). The term “interaction” was referred to as a specific and purposeful associative event under biomolecular forces between two molecules according to the definition given by Sharma et al. [50]. Finally, articles that did not provide additional information to papers previously published and included in the scoping review were eliminated.

### Literature search and selection of sources of evidence

In order to identify relevant documents, two online databases were selected: PubMed and Web of Science Core Collection (WOS CC). Multiple keywords referring to helminth parasites (parasite, helminth, worm, nematode, platyhelminth, trematode, cestode) and the main components of the haemostatic system of vertebrates (haemostasis/haemostatic system, coagulation, platelet, vWF, TF, factor V, factor VII, factor VIII, factor IX, factor X, factor XI, factor XII, factor XIII, prothrombin, thrombin, fibrinogen, fibrin, TFPI, AT-III, APC, fibrinolysis/fibrinolytic system, plasminogen, plasmin, tPA, uPA, PAI-1, PAI-2, A2AP) (reviewed in [4]) combined with the Boolean operators AND/OR were selected to design the literature search strategy. Since PubMed possesses a thesaurus, MeSH (Medical Subject Heading) terms were included in the search strategy for this database. The search strategy for both databases can be found in Supplementary Methods 1. The final search results were exported to an Excel file (Microsoft Corp., Redmond, WA, USA) and duplicates were removed. The selection process of sources of evidence was carried out following the above mentioned inclusion/exclusion criteria. Two authors (A.D. and J.S.) were chosen to search and select independently the sources of evidence, requiring double approval in each step. Any disagreement that arose was resolved by consulting the corresponding author in order to avoid any risk of bias.

### Data charting process

Relevant information from the selected documents was entered in an Excel file following a standardised protocol designed for this study (Supplementary Methods 2). Information extracted from the selected publications included data on article (accession number, bibliographic reference, year of publication), parasite (species, stage, parasitic material, description of the parasitic material, protein compartment) and host–parasite interaction (type of interaction, interacting component of the host haemostatic system, interaction study technique, interacting parasite molecule identified, identification technique, interacting pathway of the host haemostatic system, effect on blood clots formation/dissolution in the host, biological process attributed to the interaction, and validation of the attributed process). Data were independently charted by two authors (A.D. and F.S.), requiring agreement between them and consulting the corresponding author if any conflict arose.

## Results

### General considerations

**Figure 1:**
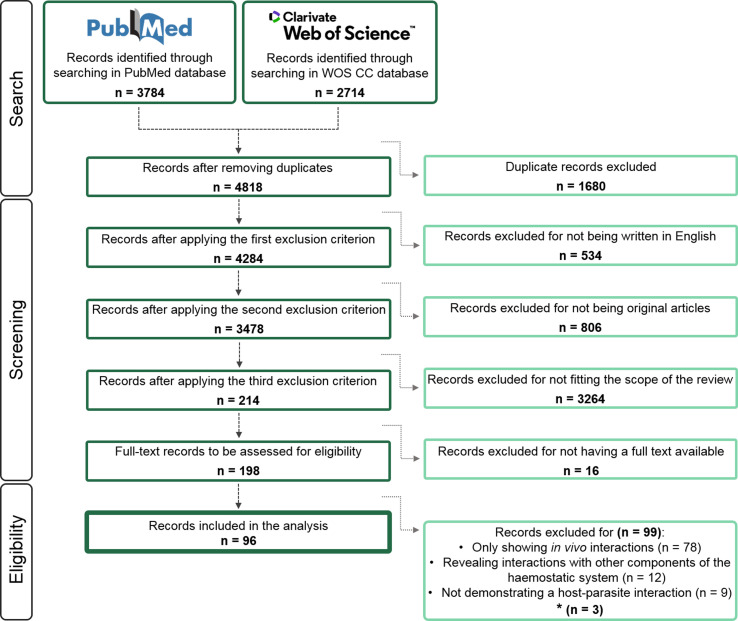
Flow diagram of the literature search and selection process. Number of publications identified through searching in each database and obtained after removing duplicates and applying each eligibility criterion. *Records eliminated for providing data that have been previously published in other articles included in the scoping review.

**Figure 2:**
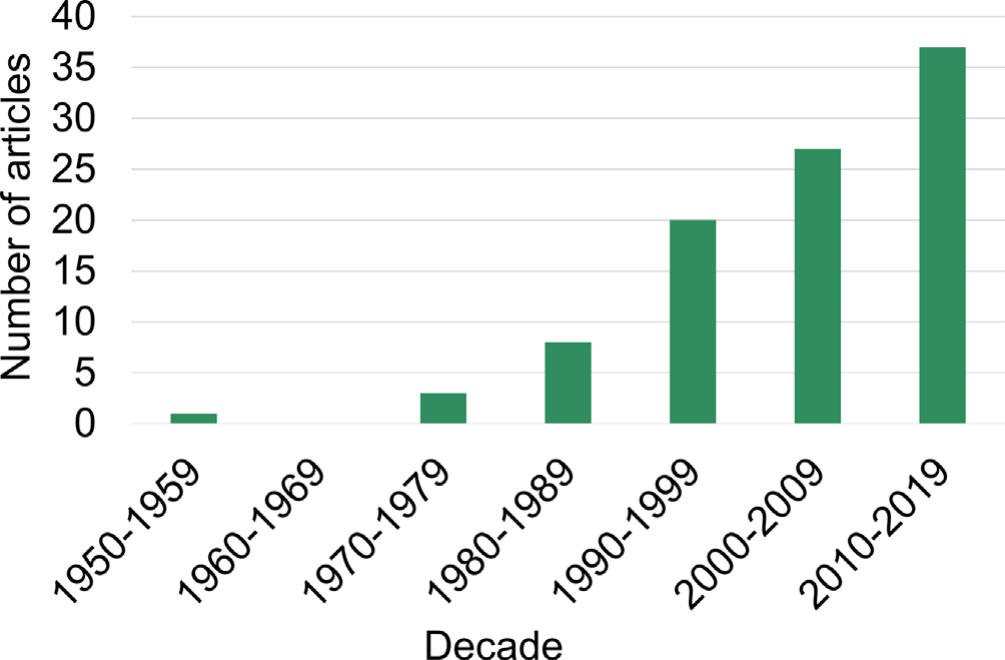
Number of sources of evidence per decade. Number of publications included in the present scoping review for their analysis per decade.

After duplicates were removed, a total of 4818 publications were screened following the eligibility criteria described in Figure 1. Of these, 96 sources of evidence (Supplementary References) were finally included in the subsequent analyses (Supplementary Data). All documents described experimental procedures carried out to demonstrate specific and purposeful associative events (binding, activation, inhibition or degradation) between helminth parasites and the abovementioned components of the haemostatic system of their vertebrate hosts. A total of 259 interactions were reported, differing in at least one of the following characteristics: parasitic material, parasite stage, parasite species and component of the host haemostatic system. The considered time period covered 64 years from 1956, when the first article identified in the present study was published, to 2019, the last year considered in this review. Only in 35 of the 64 years, at least one article related to the scope of the review was published. The rate of publication increased progressively from 32 papers published in the 20th century (spread over 16 different years) to 64 articles published in the 21st century (spread over 19 different years) (Fig. 2).

### Characteristics of the host–parasite interaction

**Figure 3:**
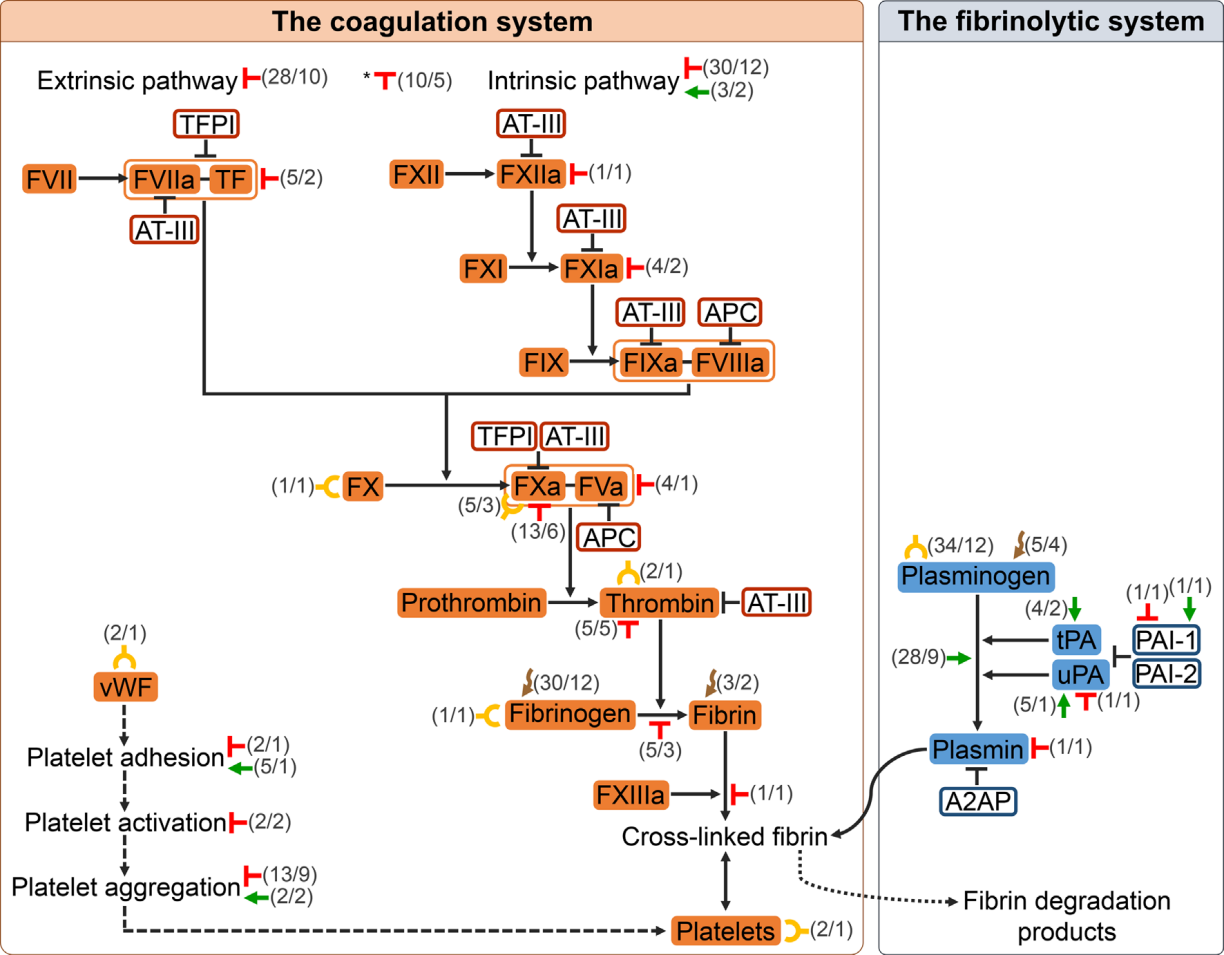
Scheme of the haemostatic system. The diagram represents the physiological haemostatic system of vertebrates. The components of the coagulation system (coloured orange) and the fibrinolytic system (coloured blue) analysed in the present study and the interactions that occur between them are shown. Two components of the coagulation system included in a box and joined by a hyphen (–) constitute a complex acting as a group. The continuous arrow, the symbol “**⊥**“ and the curved arrow between two components of the haemostatic system indicate activation, inhibition and degradation, respectively, of the second component by the first. The double arrow indicates cohesion between two components. The dashed arrow indicates different steps of a process. The diagram also represents the interactions between the species of helminth parasites and the host haemostatic system identified in the scoping review. The symbols coloured green, red, yellow and brown indicate activation, inhibition, binding and degradation, respectively, of the component of the haemostatic system marked by at least one of the species of helminth parasites analysed. These symbols are accompanied by two numbers in parenthesis separated by a slash: (the number of interactions of the specific type of interaction represented/the number of species of helminth parasites in which the type of interaction was described). (*) Interactions resulting in inhibition of coagulation without specifying the pathway. Abbreviations: FVII: factor VII; FVIIa: activated factor VII; TF: tissue factor; TFPI: tissue factor pathway inhibitor; AT-III: antithrombin III; FXII: factor XII; FXIIa: activated factor XII; FXI: factor XI; FXIa: activated factor XI; FIX: factor IX; FIXa: activated factor IX; FVIIIa: activated factor VIII; APC: activated protein C; FX: factor X; FXa: activated factor X; FVa: activated factor V; FXIIIa: activated factor XIII; vWF: von Willebrand factor; tPA: tissue plasminogen activator; uPA: urokinase-type plasminogen activator; PAI-1: plasminogen activator inhibitor 1; PAI-2: plasminogen activator inhibitor 2; A2AP: α2-antiplasmin.

Most of the interactions identified in the present study between helminth parasites and the host haemostatic system involved components of the coagulation system (179 interactions, 69.11%). Out of these, 76 interactions (42.46%) occurred with different pathways of the coagulation cascade, 31 (17.32%) with fibrinogen, 26 (14.53%) with platelets and 18 (10.06%) with FXa. The number of interactions with other components of the coagulation system [vWF, activated factor VII/tissue factor complex (FVIIa/TF), FXIIa, activated factor XI (FXIa), factor X (FX), activated factor X/activated factor V complex (FXa/FVa), thrombin and fibrin] ranged from 1 to 7. In most instances, the result of such interactions was the inhibition of the coagulation process (123 interactions, 68.72%), mainly through the inhibition of the extrinsic, intrinsic and common pathways, FXa and platelet aggregation. The remaining interactions with the coagulation system were related to its activation (10 interactions, 5.59%), the binding to platelets and different coagulation factors (13 interactions, 7.26%) and the degradation of fibrinogen and fibrin (33 interactions, 18.44%) (Fig. 3). The interaction between helminth parasites and the fibrinolytic system was described in 80 cases (30.89%), with plasminogen as the fibrinolytic molecule having the greatest number of reported interactions, a total of 67 (83.75%). Between 1 and 6 interactions were identified with other fibrinolytic components (tPA, uPA, PAI-1 and plasmin). Thirty-eight (47.50%) of these interactions were directly related to the activation of the pathway and 3 (3.75%) to its inhibition. Plasminogen binding (34 interactions, 42.50%) and plasminogen degradation (5 interactions, 6.25%) corresponded to the remaining interactions identified between helminth parasites and this system (Fig. 3).

#### Effect on blood clot formation/dissolution and biological process attributed to the host–parasite interaction

**Figure 4:**
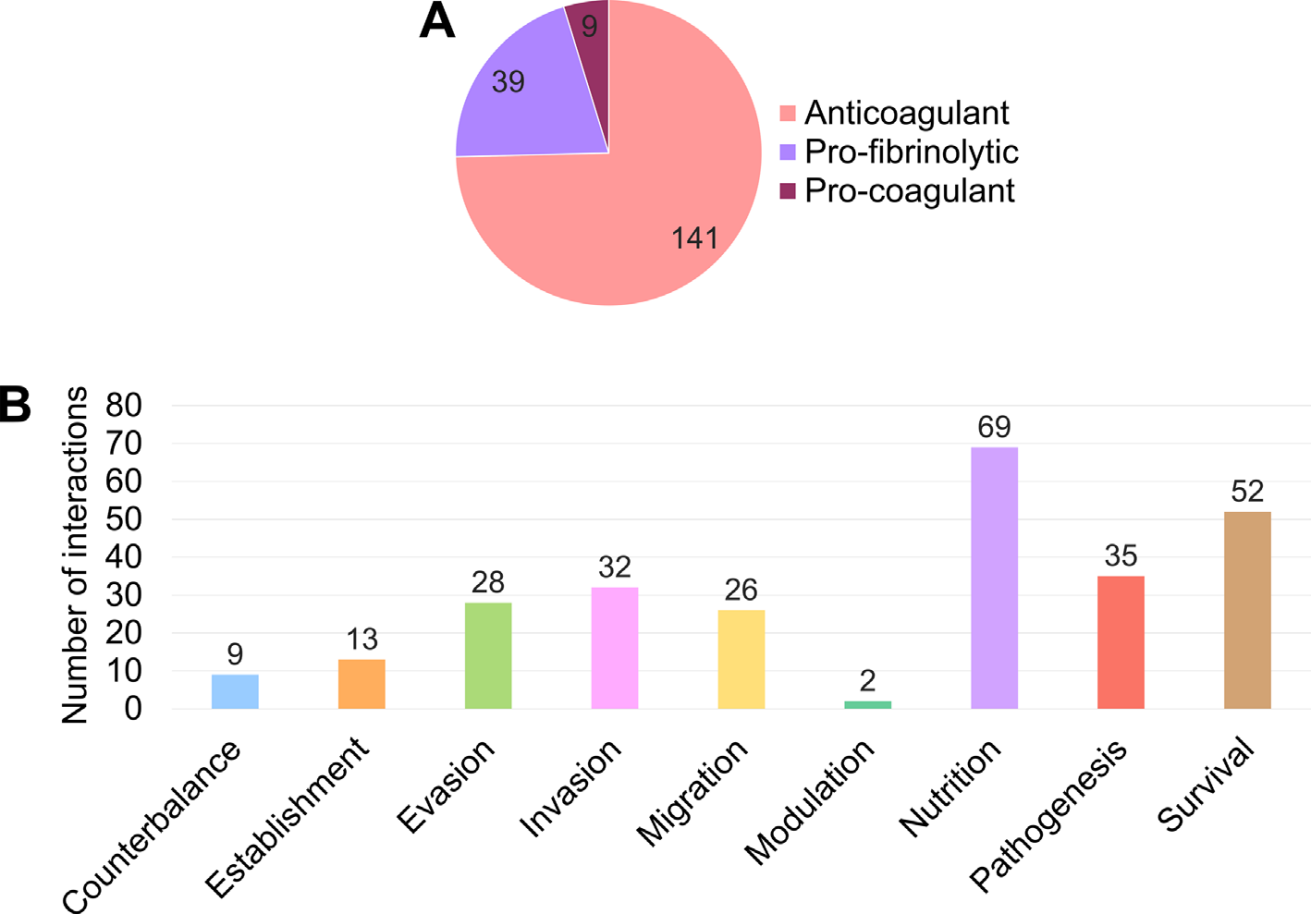
Effect on host blood clot formation/dissolution (A) and biological process (B) attributed to the host–parasite interaction. Number of interactions per effect related to the formation/dissolution of blood clots in the host (A) and number of interactions per biological process in which the interaction could be involved (B).

According to the information provided by the sources of evidence analysed, the potential effect of the interaction between helminth parasites and the haemostatic system on the formation/dissolution of blood clots in the host was described in 189 cases and classified as anticoagulant (74.60%), pro-fibrinolytic (20.63%) and pro-coagulant (4.76%) (Fig. 4A). Interactions with components of the coagulation system mostly resulted in an anticoagulant effect (92.62%), while a pro-fibrinolytic potential was predominantly attributed to interactions with the fibrinolytic system (92.50%).

The biological process in which the host–parasite interaction could be involved was indicated in 184 of the total 259 interactions analysed and it was classified as parasite nutrition (37.50%), parasite survival (28.26%), pathological mechanisms in the host (19.02%), host-tissue invasion (17.39%), evasion of host-defence systems (15.22%), migration through the host tissues (14.13%), establishment in the host (7.07%), counterbalance of the parasite to other effects caused by itself (4.89%) and modulation of host mechanisms (1.09%) (Fig. 4B). Out of these, nutrition (48.28%) was the most frequently attributed process to interactions with the coagulation system, while survival (48.53%) and pathogenesis (44.12%) were the top-two processes ascribed to interactions with the fibrinolytic system.

After comparing the potential effect of the interaction on the formation/dissolution of blood clots and the biological process in which the interaction could participate, the results showed that an anticoagulant potential was mainly related to parasite nutrition (50.54%) and a pro-fibrinolytic effect was linked to parasite survival (63.64%) and the appearance of pathological mechanisms in the host (45.45%).

Out of the 96 publications collected in the present scoping review, only 2 (2.08%) included experiments to validate that the host–parasite interaction identified was involved in the attributed biological processes described above. In particular, these studies revealed the stimulation of cell proliferation and migration and the degradation of extracellular matrix upon *Dirofilaria immitis* activation of the host fibrinolytic system (see document numbers 76 and 78 in Supplementary Data).

### Biological and molecular information about parasites

#### Parasite species

**Figure 5:**
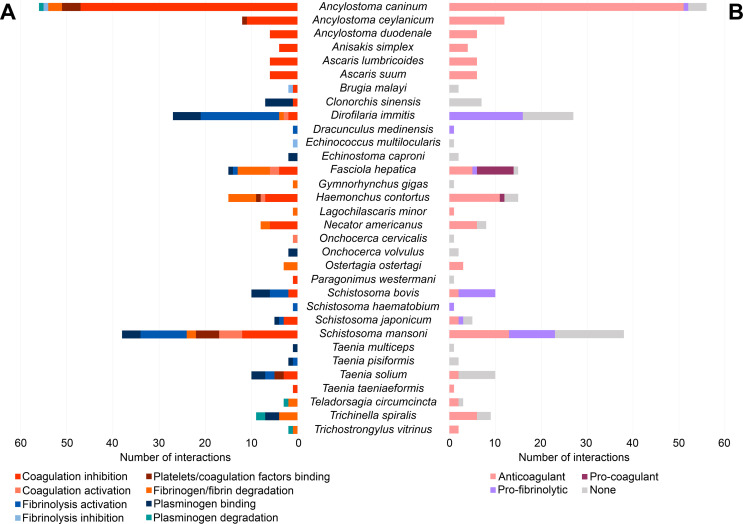
Type of interaction with the host haemostatic system (A) and effect on host blood clot formation/dissolution (B) per helminth species. Number of interactions per parasite species and type of interaction with the host haemostatic system (A) and number of interactions per parasite species to which each potential effect on blood clot formation/dissolution in the host was attributed (B).

According to the information available in the selected sources of evidence, interactions with the host haemostatic system were identified in 32 species of helminth parasites belonging to 22 genera (18 nematodes, 8 trematodes and 6 cestodes). The species that showed the highest number of interactions was *Ancylostoma caninum* (56 interactions, 21.62%), followed by *Schistosoma mansoni* (38 interactions, 14.67%) and *D. immitis* (27 interactions, 10.42%). In the remaining 29 species, between 1 and 15 interactions were described (Fig. 5).

**Figure 6:**
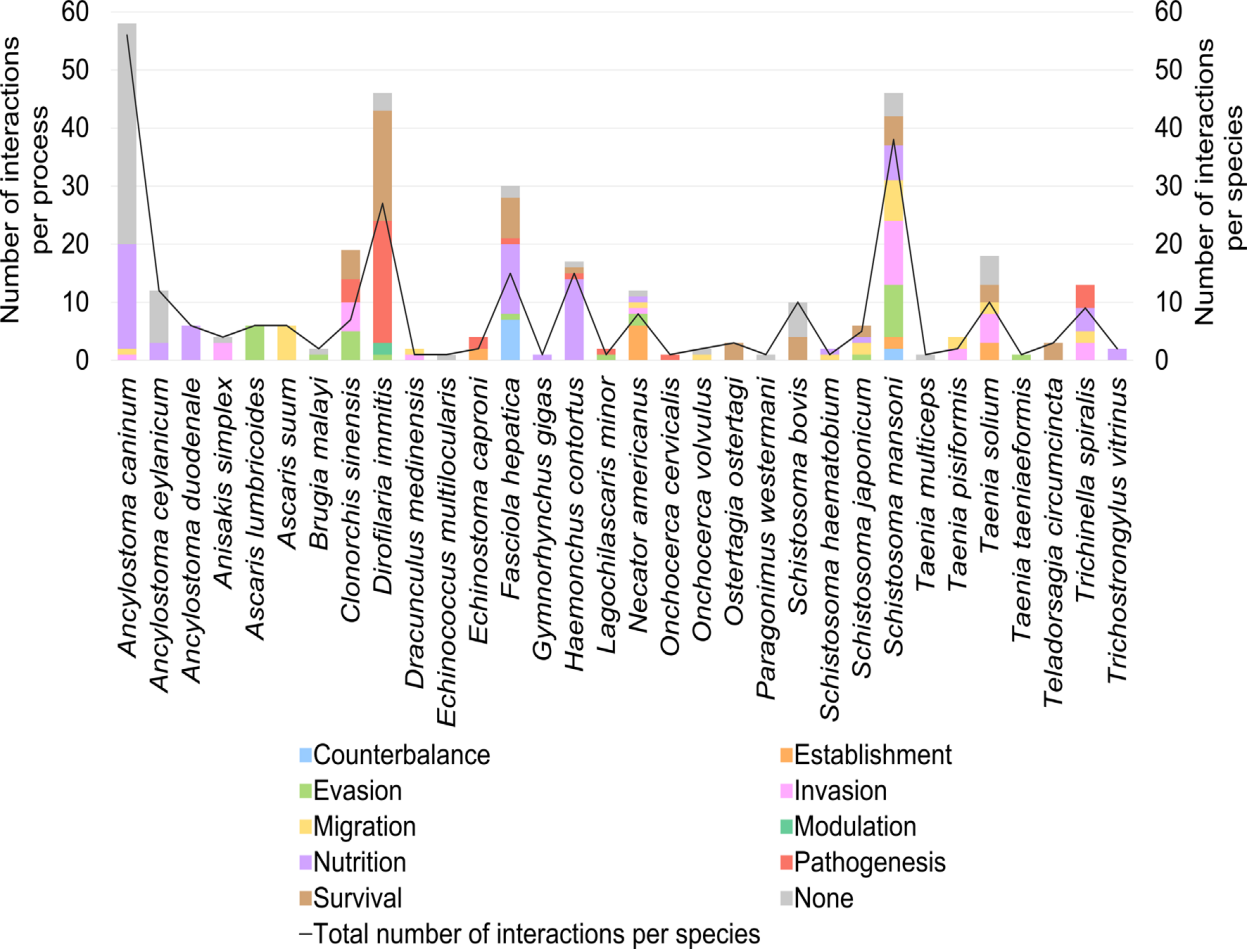
Biological process attributed to the host–parasite interaction per helminth species. Results are expressed as number of interactions per parasite species and biological process (left axis and bars) and total number of interactions with the host haemostatic system identified per parasite species (right axis and black line).

Out of the total number of 32 species, 25 (78.13%) revealed interactions with the coagulation system and 19 (59.38%) with the fibrinolytic system. For some of them, such as *A*. *caninum*, *A. ceylanicum*, *Haemonchus contortus*, *Fasciola hepatica* or *Necator americanus*, interactions with the coagulation system were primarily reported, while others, such as *Clonorchis sinensis*, *D*. *immitis* or *S. bovis*, predominantly showed interactions with the fibrinolytic system. In other species (*S. mansoni*, *Taenia solium* or *Trichinella spiralis*), a similar number of interactions was described with both haemostatic pathways. In general, the predominant interactions in which these species participated were the inhibition of the coagulation system, the activation of the fibrinolytic system, the degradation of fibrinogen and fibrin and the binding of plasminogen, coagulation factors and platelets (Fig. 5A). The specific interactions between each parasite species and the host haemostatic system are collected in Supplementary Table 1. In all species, these interactions were mainly associated with an anticoagulant and/or pro-fibrinolytic effect except for *F*. *hepatica*, which was mostly related to a pro-coagulant potential (Fig. 5B). The interactions between some species, such as *Ancylostoma* spp., *F*. *hepatica* or *H*. *contortus*, and the host haemostatic system were mainly related to parasite nutrition. In the case of *D*. *immitis*, the most attributed biological processes to its interaction with the host haemostatic system were parasite survival and the onset of pathological mechanisms in the host. Regarding *S*. *mansoni*-haemostatic system interactions, they were linked with all the above mentioned biological processes, except for pathogenesis and modulation (Fig. 6).

#### Parasite stage and parasitic material

**Figure 7:**
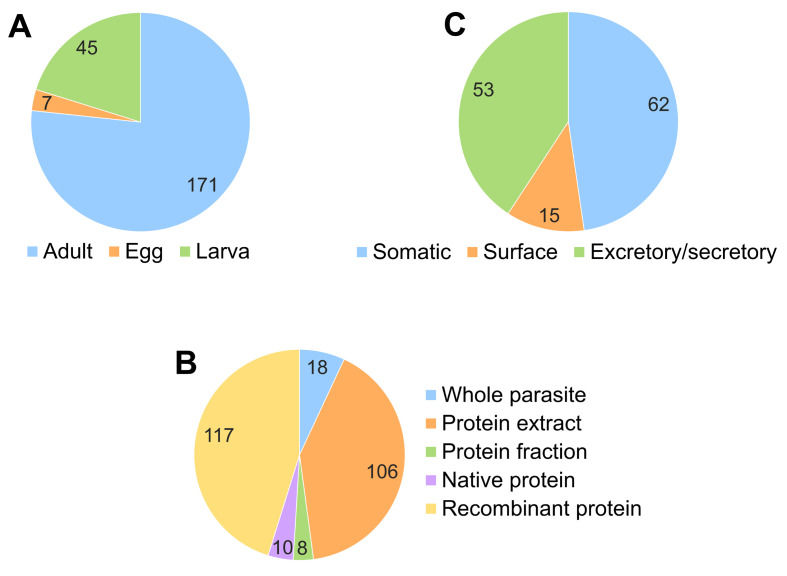
Parasite stage (A), parasitic material (B) and protein compartment (C) in which the host–parasite interaction was described. Number of interactions per parasite stage (A), parasitic material (B) and protein compartment (C) employed to study the interaction with the host haemostatic system.

Of the 259 interactions analysed, 223 reported data on the parasite stage employed to study the interaction. Out of these, 76.68% were found in adult worms, 20.18% in larval stages and 3.14% in eggs (Fig. 7A). The most attributed biological processes to the interactions described in adult parasites were nutrition (45.30%), survival (37.61%) and pathogenesis (27.35%), while the interactions identified in larval stages were mainly related to invasion (41.67%), evasion (30.56%) and migration (19.44%) mechanisms.

The two parasitic materials more frequently employed to evaluate the interactions with the host haemostatic system were recombinant proteins (45.17%) and protein extracts (40.93%). In the remaining interactions, whole parasites (6.95%), native proteins (3.86%) and protein fractions (3.09%) were used (Fig. 7B). The nature of the protein compartment used to study the interaction with the host haemostatic system was reported in 130 cases. Out of these, 47.69% of the interactions were identified in the somatic extract, 40.77% in the excretory/secretory products and 11.54% in the surface-associated molecules (Fig. 7C).

Considering the two most used parasitic materials, it was observed that protein extracts were used throughout the whole period analysed (1956–2019), while recombinant proteins were used from 1996 onwards. In the 20th century, 64.21% and 15.79% of the interactions were analysed using protein extracts and recombinant proteins, respectively. In contrast, in the 21st century, the percentage of interactions evaluated using protein extracts decreased to 27.44%, while the use of recombinant proteins increased to 62.20%.

#### Parasite molecules

Of the 259 interactions analysed in the present study, 154 reported data on the parasite molecule responsible for the interaction (either used to carry out the experiments or identified after discovering the interaction). Proteins were responsible for the interaction in all cases (152 interactions, 98.70%) except for two interactions carried out by carbohydrates (Supplementary Table 2). Fifty-three parasite proteins involved in interactions with the host haemostatic system were identified. The group that showed the highest number of interactions was that including different *Ancylostoma* spp. anticoagulant peptides/proteins (49 interactions, 32.24%), followed by enolases (20 interactions, 13.16%), serine proteases (12 interactions, 7.89%), annexins (11 interactions, 7.24%), cathepsins and metalloproteases (10 interactions per group, 6.58%), cysteine proteases and glyceraldehyde-3-phosphate dehydrogenases (GAPDH) (9 interactions per group, 5.92%), actins (8 interactions, 5.26%), aspartic proteases and fructose-bisphosphate aldolases (7 interactions per group, 4.61%), serine protease inhibitors (serpins) (6 interactions, 3.95%) and galectins and Kunitz-type proteins (5 interactions per group, 3.29%). The remaining 39 proteins showed between 1 and 3 interactions (Supplementary Table 2). Enolase was the protein identified as interacting molecule in a higher number of helminth parasite species (12 species, 37.50%), followed by serine proteases (7 species, 21.88%) and serpins (6 species, 18.75%). Aspartic proteases, cysteine proteases, metalloproteases and GAPDH were identified in 5 species (15.63%). The number of species in which the remaining 46 proteins were described as interacting molecules ranged from 1 to 3 (Supplementary Table 2).

Considering the most recurrently identified proteins, data showed that some of them revealed interactions only with components of the coagulation system (aspartic proteases, cysteine proteases and Kunitz-type proteins) or the fibrinolytic system (actins, enolases, fructose-bisphosphate aldolases, galectins and GAPDH), while in others (annexins, *Ancylostoma* spp. anticoagulant peptides/proteins, cathepsins, metalloproteases, serine proteases and serpins), interactions with both pathways were described, although interactions with the coagulation system were predominant. The majority of these proteins were only related to an anticoagulant (*Ancylostoma* spp. anticoagulant peptides/proteins, aspartic proteases, serpins, Kunitz-type proteins) or pro-fibrinolytic (actins, enolases, fructose-bisphosphate aldolases, galectins, GAPDH) potential, or both in the case of annexins (66.67% anticoagulant/33.33% pro-fibrinolytic). Other proteins were associated with an anticoagulant and/or pro-fibrinolytic potential together with a pro-coagulant effect, such as cathepsins (62.50% anticoagulant/12.50% pro-fibrinolytic/25.00% pro-coagulant), cysteine proteases (57.14% anticoagulant/42.86% pro-coagulant), metalloproteases (70.00% anticoagulant/30.00 pro-coagulant) and serine proteases (33.33% anticoagulant/33.33% pro-fibrinolytic/33.33% pro-coagulant).

### Techniques

According to the data obtained in the present scoping review, the most frequently employed techniques to evaluate the interaction between helminth parasites and the coagulation system were clotting time assays (76 interactions, 42.46%), SDS-PAGE (22 interactions, 12.29%) and chromogenic assays (15 interactions, 8.38%). The most frequently measured coagulation times were the activated partial thromboplastin time (APTT) (34 interactions, 18.99%), the prothrombin time (PT) (28 interactions, 15.64%) and the thrombin time (TT) (5 interactions, 2.79%), which were employed to study the intrinsic (APTT), extrinsic (PT) and common pathways (APTT and PT) of the coagulation cascade and the conversion of fibrinogen into fibrin (TT). The SDS-PAGE and the chromogenic assays were used to evaluate the binding/degradation and the inhibition of coagulation factors by parasites, respectively. In the case of the fibrinolytic system, interactions of helminth parasites with this pathway were mainly studied through blot assays (33 interactions, 41.25%), chromogenic assays (24 interactions, 30.00%) and ELISA (22 interactions, 27.50%). Blot assays and ELISA were employed to study plasminogen binding and expression of the plasminogen activators/inhibitors, while chromogenic assays were used to study plasminogen activation. In 71 of the total 179 interactions identified with the coagulation system (39.66%), and in 13 of the total 80 interactions identified with the fibrinolytic system (16.25%), 33 and 6 techniques different from those mentioned above were used, respectively.

Of the 132 interactions identified in protein extracts/whole parasites/protein fractions, 27 (20.45%) showed data on the identification of the molecule(s) responsible for the interaction. The techniques mostly employed to identify these molecules were SDS-PAGE (10 interactions, 37.04%) and mass spectrometry (5 interactions, 18.52%).

## Discussion

Helminth parasites are among the most common groups of infectious agents of both humans and animals around the world. Diseases caused by these pathogens, known as helminthiases, affect more than one billion people living mainly in poverty in tropical and sub-tropical areas, causing devastating health, social and economic problems [60]. In addition, helminthiases account for more than half of all farm diseases, which generate a negative impact on animal welfare, a reduction in the yield of animal-derived sub-products, and overall result in huge economic losses for the livestock industry worldwide [47]. However, and despite their importance, the control of helminthiases is still challenging and currently based on drug treatment [60]. Additionally, the fact that anthelmintic resistance is emerging in many species [13], together with the severe consequences of these parasitoses, suggests the need to develop new control strategies, such as vaccination, for which unravelling host–parasite interactions is essential [23, 47]. Among these interactions, the molecular relationships between helminth parasites and the haemostatic system of their vertebrate hosts have been studied in recent decades [5, 16, 22, 30, 39, 41, 61], but the evidence is extensive and broadly dispersed within published articles. In accordance with this, the objective of the present work was to carry out a scoping review in order to systematically summarize and update the published evidence and concepts about this topic, thus contributing to future research in this field.

The analyses showed a notable number of blood and tissue-helminth parasite species with capability to interact with the haemostatic system of their vertebrate hosts by means of similar strategies. This is suggested by the obtained results, which showed that some common molecules are used by different species of parasites to manipulate the same components of the host haemostatic system. The fact that species belonging to such distantly related taxa [29] and parasitizing a wide variety of vertebrate hosts share similar mechanisms to interact with the haemostatic system denotes their importance and evolutionary convergence, as has been postulated for this and other parasitic adaptations [22, 25, 36, 46, 61]. Besides helminths, protozoan and arthropod parasites and even bacteria and fungi exploit the host haemostatic system [5, 12], which supports the abovementioned assertion in terms of evolution and significance.

The obtained results also revealed a high number of different parasite proteins with capability to interact with the host haemostatic system, many of them involved in the same interactions. This suggests that parasites employ different molecules aimed at accomplishing the same function [22, 37, 49], which is considered as one of the main reasons behind the challenge of vaccine development against helminthiases [37]. Conversely, it was also observed that, in many cases, the same protein interacted with different components of the host haemostatic system. It is worth mentioning that the vast majority of the reported proteins have been described as having other canonical functions, apart from their participation in the manipulation of the host haemostatic system. This mechanism is referred to as “moonlighting” and constitutes an advantageous parasite strategy of energy saving based on proteins exhibiting “expected or unexpected” functions depending on some factors, such as their different location or secretory pathways [7, 26]. In line with this, our results showed that the majority of the interactions were described in surface parasitic extracts and excretory/secretory products, which indicates that parasite molecules involved in the exploitation of the host haemostatic system are mainly expressed on the parasite surface or secreted to their environment from the cytosol. This location at the host–parasite interface could favour their interaction with host molecules and allow parasites to benefit from the result of the interaction in their immediate habitat or in those sites where it is required.

The term “moonlighting proteins” has been specially attributed to proteins that interact with the fibrinolytic system, specifically to those whose main role is acting as catalytic enzymes in the glycolytic process [16, 18, 22, 27]. Among these proteins, enolase, GAPDH and fructose-bisphosphate aldolase were the most recurrent plasminogen receptors identified in this scoping review, in addition to other less frequently identified proteins, such as phosphoglycerate mutase and triose phosphate isomerase. These proteins are not only widely extended as plasminogen receptors among helminth parasites, but also among protozoan parasites, bacteria and fungi [5]. Other proteins identified in this review as plasminogen-binding proteins with canonical functions not related to glycolysis were actin and annexin, among others. Intriguingly, in addition to fibrin of blood clots, plasminogen of vertebrates can bind to receptors located on the surface of a wide variety of cells, among which are α-enolase and annexin 2 [10]. Therefore, it seems that parasites could use similar molecules to those expressed as physiological receptors in vertebrate hosts as plasminogen receptors. In line with this, it has been postulated that plasminogen-binding mechanisms are similar to both host and parasite receptors [16]. These mechanisms are commonly mediated by the presence of carboxy-terminal lysine residues in the host and parasite receptors with the ability to interact with lysine-binding sites within the kringle domains of plasminogen [40]. This feature can be experimentally demonstrated by performing competition assays with lysine analogues, such as ε-aminocaproic acid. Even though the term “moonlighting proteins” has not yet been linked with parasite molecules that interact with the coagulation system, some of the proteins reported in the present scoping review that interacted with this pathway of the haemostatic system, such as annexin, calpain, calreticulin or ectonucleotide pyrophosphatase/phosphodiesterase, have other canonical functions within the parasite physiology that are unrelated to the interaction with the host blood coagulation [57]. Other proteins (e.g., *Ancylostoma* spp. anticoagulant peptides/proteins, Kunitz-type proteins and serpins), which accounted for most interactions with the coagulation system, exhibit serine-type endopeptidase inhibitor activity [41, 44]. This is noteworthy given that most proteins participating in blood coagulation in vertebrates are serine proteases and their main inhibitors (AT-III and TFPI) are a serine protease inhibitor (serpin) and a Kunitz-type inhibitor, respectively [4]. Moreover, protein-sequence comparison studies between nematode and mammalian serpins have highlighted that both share some of the key amino acids to maintain the structure and function of the proteins [61]. Nevertheless, further investigation is needed to determine whether this is the case for the helminth serpins involved in the manipulation of the host haemostatic system.

As reported by the authors of the selected publications, interactions between helminth parasites and the host haemostatic system were mainly related to the capability of parasites to prevent the formation of blood clots (anticoagulant potential) and facilitate their dissolution (pro-fibrinolytic potential). The analysis revealed that these parasite strategies were mainly associated with their nutrition requirements and survival mechanisms, respectively. Consequently, these interactions could be especially beneficial for blood feeding parasites, such as the adult stages of *Ancylostoma* spp., *F*. *hepatica* or *H*. *contortus*, as well as for blood parasites (e.g., *D. immitis* or *Schistosoma* spp.), respectively. In fact, it has been suggested that the formation of blood clots in the host could constitute a physical barrier for parasites that migrate through host tissues, live in the circulatory system or feed on blood [22]. Moreover, the manipulation of the host haemostatic system could provide parasites with additional benefits other than avoiding blood clot formation. This is because the coagulation system is also considered an important defensive mechanism that is activated during infections and some of its components are involved in the immune response and immune system modulation [3]. Regarding fibrinolysis, plasmin could also play an important role in the evasion and modulation of host responses, since it exerts its proteolytic activity against immunoglobulins and complement components besides fibrin of blood clots, as has been shown in some species of pathogenic bacteria [35, 51, 59]. Plasmin also can directly degrade components of the extracellular matrix and activate some matrix metalloproteinases, both resulting in the degradation of the extracellular matrix [55]. Additionally, plasmin is involved in cell proliferation and migration and the activation of some angiogenic factors, participating in the pathogenesis of cancer and several diseases with an inflammatory component [6, 31, 43]. Thus, the activation of the plasminogen/plasmin system by bacteria has also been attributed to adhesion, invasion and migration processes [8, 15, 54] and degradation of host proteins for nutrition [28]. These are likely the reasons why the interactions identified in the present scoping review were mostly related by the authors of the publications to parasite survival mechanisms, namely nutrition, invasion of host tissues, evasion of host responses and migration as well as to the appearance of pathological processes in the host. Nevertheless, the physiological significance of these interactions in helminthiases has not yet been fully demonstrated since only 2.08% of the studies reviewed in the present work included validation experiments to determine whether the interaction was indeed involved in the presumably related biological processes. The study of the biological significance of these mechanisms could provide valuable information for a better understanding of the real role of the interaction between parasites and the host haemostatic system, as has been shown for the nematode *D*. *immitis* [20–22] and some protozoan parasites [2, 38].

Regarding the parasitic stage in which the interactions with the host haemostatic system were described, these were reported from adults to eggs and different larval stages. These results indicate that the host haemostatic system could be exploited by helminth parasites throughout their whole intra-vertebrate life cycle, as observed in the trematode *S*. *mansoni* (Supplementary Data). As regards the protozoan parasites *Trypanosoma* spp. and *Plasmodium* spp., it has been demonstrated that stages that develop in vectors are able to co-opt proteins from the vertebrate blood meal to favour their transmission to the vertebrate host and/or the invasion of vector tissues [2, 17, 48, 58]. It remains to be determined whether something similar occurs in the context of vector-borne transmitted helminths. The fact that most interactions were identified in adult worms indicates that this is the favourite parasitic stage employed to study interactions between helminth parasites and the host haemostatic system. Using larval stages (preferably early stages) could contribute to increase the knowledge of the functioning and implication of these interactions at the beginning of the infection in helminthiases, a key point for the establishment of the parasite in the host. Unravelling host–parasite molecular interactions at this critical moment is essential to develop effective control strategies against parasites [23].

According to the results obtained in this work, a wide variety of methodologies were employed to study helminth parasite interactions with the host haemostatic system, mainly ELISA, SDS-PAGE, blot assays, chromogenic assays and coagulation time assays. The use of other tests scarcely identified in the present scoping review and routinely used in clinical practice, such as thromboelastography (only employed by the study conducted by Da’dara et al. [14]), would be encouraged to study these interactions since they allow us to simultaneously analyse different parameters of haemostasis [45]. Similarly, the use of novel techniques to identify the molecule(s) responsible for the interaction, such as -omics approaches, little identified in this scoping review, could be useful to better understand host–parasite relationships and find new and effective therapeutic targets [23, 47, 53]. In fact, some of the parasite proteins reported in the present scoping review and identified as interactors with the host haemostatic system (mainly serpins and “moonlighting proteins”) have been postulated as potential targets for vaccination or anthelmintic drugs [16, 22, 61]. In addition, a deeper study of these parasite molecules and their functions could also lead to the development of new drugs for human therapy in plasmin-induced pathologies or haemostatic disorders. In line with this, the nematode anticoagulant protein c2 from *A*. *caninum* (NAPc2) (see Supplementary Data) has been tested as an antithrombotic agent in phase II clinical studies in humans with promising results, since it was found to be a safe, well-tolerated and effective molecule to reduce thrombus formation in coronary complaints or during and after operations [19, 32, 42]. Recently, this protein has also been proposed as a candidate for evaluation in patients hospitalized with COVID-19 at elevated risk for thrombosis [24].

Finally, it is worth highlighting that the high number of reported interactions in some helminth species, such as *A. caninum*, *S. mansoni* or *D. immitis*, is unlikely biologically relevant. Presumably, this high number of reported interactions specifically in these parasite species is rather related to the close contact that these parasites have with the host cardiovascular system, to their use as parasitic models or to the scientific interest that they have sparked in some research groups. Along with this bias, our scoping review has certain limitations arising from the protocol designed and the literature search. These criteria were chosen in order to make our review more feasible, but other factors potentially influencing our conclusions should be taken into account, such as language, the type of article or the date range selected.

## Conclusions

To conclude, the present scoping review highlights the importance of the exploitation of the host haemostatic system by helminth parasites by bringing to light the great number of different parasitic species with capability to utilise this mechanism throughout their different life stages. The huge parasitic repertoire of proteins that are able to interact with a high number of components of this host molecular pathway, and their homology with the physiological receptors/activators/inhibitors of their hosts, reflects the biochemical redundancy of this parasite mechanism, as well as the difficulty to confront it from the point of view of therapeutic control. Despite the complexity, the obtained results also showed a common pattern of interaction between different helminth parasite species and the haemostatic system of their hosts, suggesting that such an event appeared by evolutionary convergence. Parasites could benefit from this manipulation in terms of nutrition, establishment and survival within the vertebrate organism, but it can also account for the appearance of pathological mechanisms in the host. Despite growing interest in studying the interactions between helminth parasites and the host haemostatic system, knowledge gaps that have been highlighted throughout the review still remain. Unravelling the interactions of the aforementioned and other parasite species with all the components of the host haemostatic system and other interrelated systems is essential, and so is the identification of the molecules involved in these processes, which could be facilitated by means of new technical approaches. Furthermore, it is of paramount importance to determine the biological significance of these host–parasite interactions in a real physiological setting and figure out the possible applications as therapeutic targets of the molecules identified.

## Supplementary materials

Supplementary material is available at https://www.parasite-journal.org/10.1051/parasite/2022034/olm *Supplementary Checklist*. Preferred Reporting Items for Systematic reviews and Meta-Analyses extension for Scoping Reviews (PRISMA-ScR) Checklist. *Supplementary Methods 1*. Literature search strategy for each database employed to search for the sources of evidence included in the scoping review. *Supplementary Methods 2*. Protocol designed to extract relevant information from the sources of evidence included in the scoping review. *Supplementary References*. Full references of the sources of evidence included in the scoping review. *Supplementary Data*. Relevant information extracted from the sources of evidence included in the scoping review. *Supplementary Table 1*. Interactions between each helminth parasite species and the host haemostatic system. *Supplementary Table 2*. Helminth species, stage and protein compartment in which each interacting parasite molecule was identified and type of interaction with the haemostatic system.
