# Supplementary material for: Interaction of helminth parasites with the haemostatic system of their vertebrate hosts: a scoping review
Source: Parasite. 2022 Jul 14;29:35. doi: 10.1051/parasite/2022034 (PMC9281497; doi:10.1051/parasite/2022034)
Supplement: Supplementary file 2 — Supplementary Methods 1. Literature search strategy for each database employed to search for the sources of evidence included in the scoping review. [file parasite-29-35-s2.pdf]

Supplementary Methods 1. Interaction of helminth parasites with the haemostatic system of their vertebrate hosts: a scoping review. Alicia Diosdado, Fernando Simón, Judit Serrat, Javier González-Miguel. Parasite.

PubMed Search Strategy (literature search performed on 5th May 2021)

#1 "parasites"[MeSH Terms] OR "parasite*"[All Fields] OR "parasiti*"[All Fields]

#2 "helminths"[MeSH Terms] OR "helminth*"[All Fields]

#3 "worm*"[All Fields]

#4 "nematoda"[MeSH Terms] OR "nematod*"[All Fields]

#5 "platyhelminths"[MeSH Terms] OR "platyhelminth*"[All Fields]

#6 "trematoda"[MeSH Terms] OR "trematod*"[All Fields]

#7 "cestoda"[MeSH Terms] OR "cestod*"[All Fields]

#8 #1 OR #2 OR #3 OR #4 OR #5 OR #6 OR #7

#9 "hemostasis"[MeSH Terms] OR "hemosta*"[All Fields] OR "haemosta*"[All Fields]

#10 "blood coagulation"[MeSH Terms] OR "coagulation"[All Fields]

#11 "blood platelets"[MeSH Terms] OR "platelet*"[All Fields]

#12 "von willebrand factor"[MeSH Terms] OR "von willebrand factor"[All Fields]

#13 "thromboplastin"[MeSH Terms] OR "tissue factor"[All Fields]

#14 "factor v"[MeSH Terms] OR "factor v"[All Fields]

#15 "factor vii"[MeSH Terms] OR "factor vii"[All Fields]

#16 "factor viii"[MeSH Terms] OR "factor viii"[All Fields]

#17 "factor ix"[MeSH Terms] OR "factor ix"[All Fields]

#18 "factor x"[MeSH Terms] OR "factor x"[All Fields]

#19 "factor xi"[MeSH Terms] OR "factor xi"[All Fields]

#20 "factor xii"[MeSH Terms] OR "factor xii"[All Fields]

#21 "factor xiii"[MeSH Terms] OR "factor xiii"[All Fields]

#22 "prothrombin"[MeSH Terms] OR "prothrombin*"[All Fields]

#23 "thrombin"[MeSH Terms] OR "thrombin*"[All Fields]

#24 "antithrombin iii"[MeSH Terms] OR "antithrombin"[All Fields]

#25 "protein c"[MeSH Terms] OR "protein c"[All Fields]

#26 "fibrinogen"[MeSH Terms] OR "fibrin"[MeSH Terms] OR "fibrinolysis"[MeSH Terms] OR "fibrinolysin"[MeSH Terms] OR "fibrin*"[All Fields]

#27 "plasminogen"[MeSH Terms] OR "tissue plasminogen activator"[MeSH Terms] OR "urokinase type plasminogen activator"[MeSH Terms] OR "urokinase"[All Fields] OR "plasminogen activator inhibitor 1"[MeSH Terms] OR "plasminogen activator inhibitor 2"[MeSH Terms] OR "plasmin*"[All Fields]

#28 "alpha 2 antiplasmin"[MeSH Terms] OR "antiplasmin"[All Fields]

#29 #9 OR #10 OR #11 OR #12 OR #13 OR #14 OR #15 OR #16 OR #17 OR #18 OR #19 OR #20 OR #21 OR #22 OR #23 OR #24 OR #25 OR #26 OR #27 OR #28

#30 #8 AND #29

#31 #30 AND 1900/01/01:2019/12/31[Date - Publication]

Web of Science Core Collection Search Strategy (literature search performed on 5th May 2021)

#1 ALL=(parasite* OR parasiti*)

#2 ALL=(helminth* OR worm* OR nematod* OR platyhelminth* OR trematod*OR cestod*)

#3 #1 OR #2

#4 ALL=(hemosta* OR haemosta*)

#5 ALL=("coagulation" OR platelet* OR "von willebrand factor" OR "tissue factor" OR "factor v" OR "factor vii" OR "factor viii" OR "factor ix" OR "factor x" OR "factor xi" OR "factor xii" OR "factor xiii" OR prothrombin* OR thrombin* OR "antithrombin" OR "protein c" OR fibrin* OR plasmin* OR "urokinase" OR "antiplasmin")

#6 #4 OR #5

#7 #3 AND #6

Indexes=SCI-EXPANDED, ESCI Timespan=1900-2019
