# Supplementary material for: Interaction of helminth parasites with the haemostatic system of their vertebrate hosts: a scoping review
Source: Parasite. 2022 Jul 14;29:35. doi: 10.1051/parasite/2022034 (PMC9281497; doi:10.1051/parasite/2022034)
Supplement: Supplementary file 3 — Supplementary Methods 2. Protocol designed to extract relevant information from the sources of evidence included in the scoping review. [file parasite-29-35-s3.pdf]

Supplementary Methods 2. Interaction of helminth parasites with the haemostatic system of their vertebrate hosts: a scoping review. Alicia Diosdado, Fernando Simón, Judit Serrat, Javier González-Miguel. Parasite.

**ARTICLE INFORMATION**	**Document number**	Article number (in numerical order from the oldest article analysed to the most recently published)
	**Accession number**	PubMed (PMID) or Web of Science Core Collection (WOS: accession number) identifier of the article analysed
	**Bibliographic reference**	Abbreviated bibliographic reference of the article analysed
	**Year of publication**	Year of publication of the article analysed
**PARASITE INFORMATION**	**Species**	Helminth parasite species responsible for the interaction described
	**Stage**	Parasite stage used to study the interaction described. It will be defined as ADULT, EGG or LARVA. In case of the latter, it will be indicated in parenthesis the concrete larval stage of the parasite. If experiments described in the article reveal the location of the molecule responsible for the interaction in additional parasite stage(s) (after "/") to those in which the interaction is found (before "/"), both data will be separated by "/"
	**Parasitic material**	Type of parasitic material used to study the interaction described. It will be defined as NATIVE PROTEIN, PROTEIN EXTRACT, PROTEIN FRACTION, RECOMBINANT PROTEIN or WHOLE PARASITE
	**Description of the parasitic material**	Description of the parasitic material used to study the interaction described. It will be defined as the name of the native protein, protein extract, protein fraction, recombinant protein or whole parasite indicated in the article
	**Protein compartment**	Protein compartment in which the interaction is described. It will be defined as EXCRETORY/SECRETORY, SOMATIC, SURFACE or WHOLE PARASITE. If experiments described in the article reveal the location of the molecule responsible for the interaction in additional protein compartment(s) (after "/") to those in which the interaction is found (before "/"), both data will be separated by "/"
**HOST-PARASITE INTERACTION INFORMATION**	**Type of interaction**	Effect of the parasitic material employed to study the interaction on the component of the host haemostatic system analysed. It will be defined as it is described in the article
	**Interacting component of the host haemostatic system**	Molecule or step of the host haemostatic system to which the parasitic material interacts. It will be defined as it is described in the article
	**Interaction study technique**	Technique(s) employed to demonstrate the interaction between the parasitic material and the component of the host haemostatic system. It will be defined as it is described in the article
	**Interacting parasite molecule identified**	In case of protein extracts, protein fractions and whole parasites, parasite molecule(s) identified in these parasitic materials as responsible for the interaction with the host haemostatic system. It will be defined as it is described in the article
	**Identification technique**	In case of protein extracts, protein fractions and whole parasites, technique(s) employed to identify the molecule(s) responsible for the interaction with the host haemostatic system in these parasitic materials. It will be defined as it is described in the article
	**Interacting pathway of the host haemostatic system**	Pathway of the host haemostatic system to which the parasitic material interacts. It will be defined as COAGULATION or FIBRINOLYSIS
	**Effect on blood clot formation/dissolution in the host**	Potential effect attributed by the authors of the publication to the interaction in relation to the formation/dissolution of blood clots in the host. It will be defined as ANTICOAGULANT, PRO-COAGULANT, ANTI-FIBRINOLYTIC or PRO-FIBRINOLYTIC
	**Biological process attributed to the**	Biological process(es) in which the interaction could participate according to the authors of the publication. It will be defined as

	**interaction**	COUNTERBALANCE, ESTABLISHMENT, EVASION, INVASION, MIGRATION, MODULATION, NUTRITION, PATHOGENESIS or SURVIVAL. The concrete process(es) described in the article will be indicated in parenthesis
	**Validation of the attributed process**	When the participation of the interaction in the biological process(es) attributed by the authors of the publication is experimentally validated. It will be defined as YES or NO. The concrete function(s) demonstrated will be indicated in parenthesis

All the Excel cells that appear empty in Supplementary Data correspond to data that could not be extracted from the analysed publications. The symbol "—" was used to reflect this lack of information in those cases in which other data had to be inserted in the same cell (when the symbol "/" was used) or when data to be entered did not correspond to the cell in question.
