# Supplementary material for: Interaction of helminth parasites with the haemostatic system of their vertebrate hosts: a scoping review
Source: Parasite. 2022 Jul 14;29:35. doi: 10.1051/parasite/2022034 (PMC9281497; doi:10.1051/parasite/2022034)
Supplement: Supplementary file 4 — Supplementary References. Full references of the sources of evidence included in the scoping review. [file parasite-29-35-s4.pdf]

Supplementary References. Interaction of helminth parasites with the haemostatic system of their vertebrate hosts: a scoping review. Alicia Diosdado, Fernando Simón, Judit Serrat, Javier González-Miguel. Parasite.

1. Thorson RE. The effect of extracts of the amphidial glands, excretory glands, and esophagus of adults of *Ancylostoma caninum* on the coagulation of dog's blood. J Parasitol. 1956 Feb;42(1):26-30. PMID: 13295887.

2. Devi CS, Murthy DP, Devi SL, Reddy CR. Fibrinolytic activity of infective larvae of *Dracunculus medinensis*. Am J Trop Med Hyg. 1971 Jan;20(1):101-3. doi:10.4269/ajtmh.1971.20.101. PMID: 5567739.

3. Spellman GG Jr, Nossel HL. Anticoagulant activity of dog hookworm. Am J Physiol. 1971 Apr;220(4):922-7. doi: 10.1152/ajplegacy.1971.220.4.922. PMID: 5102508.

4. Tsang VC, Hubbard WJ, Damian RT. Coagulation factor XIIa (activated Hageman factor) inhibitor from adult *Schistosoma mansoni*. Am J Trop Med Hyg. 1977 Mar;26(2):243-7. doi: 10.4269/ajtmh.1977.26.243. PMID: 848647.

5. Hammerberg B, Dangler C, Williams JF. *Taenia taeniaeformis*: chemical composition of parasite factors affecting coagulation and complement cascades. J Parasitol. 1980 Aug;66(4):569-76. PMID: 6775068.

6. Greene CE, Prestwood K, Tsang VC. Effect of *Haemonchus contortus* extracts on coagulation of ovine plasma. J Parasitol. 1981 Oct;67(5):730-1. PMID: 7299587.

7. Crawford GP, Howse DJ, Grove DI. Inhibition of human blood clotting by extracts of *Ascaris suum*. J Parasitol. 1982 Dec;68(6):1044-7. PMID: 7175610.

8. Hotez PJ, Cerami A. Secretion of a proteolytic anticoagulant by *Ancylostoma* hookworms. J Exp Med. 1983 May 1;157(5):1594-603. doi: 10.1084/jem.157.5.1594. PMID: 6343546; PMCID: PMC2186995.

9. McConnell LA, Leid RW. Aggregation of equine platelets by *Onchocerca cervicalis* collagen. Am J Pathol. 1983 Mar;110(3):290-6. PMID: 6299106; PMCID: PMC1916174.

10. Boreham PF. Activation *in vitro* of some biological systems by extracts of adult worms and microfilariae of *Dirofilaria immitis*. J Helminthol. 1984 Sep;58(3):207-12. doi: 10.1017/s0022149x00026985. PMID: 6542116.

11. Carroll SM, Howse DJ, Grove DI. The anticoagulant effects of the hookworm, *Ancylostoma ceylanicum*: observations on human and dog blood *in vitro* and infected dogs *in vivo*. Thromb Haemost. 1984 Apr 30;51(2):222-7. PMID: 6740554.

12. Tamashiro WK, Rao M, Scott AL. Proteolytic cleavage of IgG and other protein substrates by *Dirofilaria immitis* microfilarial enzymes. J Parasitol. 1987 Feb;73(1):149-54. PMID: 3553516.

13. Malla N, Sofi BA, Ganguly NK, Mahajan RC. In vitro effect of larval stages of *Ascaris lumbricoides* on human blood clotting. J Helminthol. 1991 Jun;65(2):133-40. doi: 10.1017/s0022149x00010592. PMID: 1880387.

14. Liu LX, Weller PF. Intravascular filarial parasites inhibit platelet aggregation. Role of parasite-derived prostanoids. J Clin Invest. 1992 Apr;89(4):1113-20. doi: 10.1172/JCI115691. PMID: 1313445; PMCID: PMC442967.

15. Cappello M, Clyne LP, McPhedran P, Hotez PJ. *Ancylostoma* factor Xa inhibitor: partial purification and its identification as a major hookworm-derived anticoagulant *in vitro*. J Infect Dis. 1993 Jun;167(6):1474-7. doi: 10.1093/infdis/167.6.1474. PMID: 8501344.

16. Kadipasaoglu KA, Bilge FH, Baier RE. Determination of the role of cuticular carbohydrates in the hemocompatibility of *Dirofilaria immitis* (Nematoda). J Biomed Mater Res. 1993 Feb;27(2):207-16. doi: 10.1002/jbm.820270210. PMID: 8436577.

17. Knox DP, Redmond DL, Jones DG. Characterization of proteinases in extracts of adult *Haemonchus contortus,* the ovine abomasal nematode. Parasitology. 1993 May;106 (Pt 4):395-404. doi: 10.1017/s0031182000067147. PMID: 8316437.

18. Brown A, Burleigh JM, Billett EE, Pritchard DI. An initial characterization of the proteolytic enzymes secreted by the adult stage of the human hookworm *Necator americanus*. Parasitology. 1995 Jun;110 (Pt 5):555-63. doi: 10.1017/s0031182000065276. PMID: 7596639.

19. Cappello M, Vlasuk GP, Bergum PW, Huang S, Hotez PJ. *Ancylostoma caninum* anticoagulant peptide: a hookworm-derived inhibitor of human coagulation factor Xa. Proc Natl Acad Sci U S A. 1995 Jun 20;92(13):6152-6. doi: 10.1073/pnas.92.13.6152. PMID: 7597095; PMCID: PMC41660.

20. Dowd AJ, McGonigle S, Dalton JP. *Fasciola hepatica* cathepsin L proteinase cleaves fibrinogen and produces a novel type of fibrin clot. Eur J Biochem. 1995 Aug 15;232(1):241-6. doi: 10.1111/j.1432-1033.1995.tb20805.x. PMID: 7556157.

21. Rhoads ML, Fetterer RH. Developmentally regulated secretion of cathepsin L-like cysteine proteases by *Haemonchus contortus*. J Parasitol. 1995 Aug;81(4):505-12. PMID: 7623189.

22. Todorova VK, Knox DP, Kennedy MW. Proteinases in the excretory/secretory products (ES) of adult *Trichinella spiralis*. Parasitology. 1995 Aug;111 (Pt 2):201-8. doi: 10.1017/s0031182000064957. PMID: 7675535.

23. Young CJ, McKeand JB, Knox DP. Proteinases released in vitro by the parasitic stages of *Teladorsagia circumcincta*, an ovine abomasal nematode. Parasitology. 1995 May;110 (Pt 4):465-71. doi: 10.1017/s0031182000064805. PMID: 7753586.

24. Cappello M, Hawdon JM, Jones BF, Kennedy WP, Hotez PJ. *Ancylostoma caninum* anticoagulant peptide: cloning by PCR and expression of soluble, active protein in *E. coli*. Mol Biochem Parasitol. 1996 Sep;80(1):113-7. doi: 10.1016/0166-6851(96)02658-8. PMID: 8885227.

25. Furmidge BA, Horn LA, Pritchard DI. The anti-haemostatic strategies of the human hookworm *Necator americanus*. Parasitology. 1996 Jan;112 (Pt 1):81-7. doi: 10.1017/s0031182000065100. PMID: 8587805.

26. Gamble HR, Fetterer RH, Mansfield LS. Developmentally regulated zinc metalloproteinases from third- and fourth-stage larvae of the ovine nematode *Haemonchus contortus*. J Parasitol. 1996 Apr;82(2):197-202. PMID: 8604083.

27. Perteguer MJ, Raposo R, Cuéllar C. *In vitro* study on the effect of larval excretory/secretory products and crude extracts from *Anisakis simplex* on blood coagulation. Int J Parasitol. 1996 Jan;26(1):105-8. doi: 10.1016/0020-7519(95)00111-5. PMID: 9198584.

28. Stassens P, Bergum PW, Gansemans Y, Jespers L, Laroche Y, Huang S, Maki S, Messens J, Lauwereys M, Cappello M, Hotez PJ, Lasters I, Vlasuk GP. Anticoagulant repertoire of the hookworm *Ancylostoma caninum*. Proc Natl Acad Sci U S A. 1996 Mar 5;93(5):2149-54. doi: 10.1073/pnas.93.5.2149. PMID: 8700900; PMCID: PMC39925.

29. Dowd AJ, Tort J, Roche L, Ryan T, Dalton JP. Isolation of a cDNA encoding *Fasciola hepatica* cathepsin L2 and functional expression in *Saccharomyces cerevisiae*. Mol Biochem Parasitol. 1997 Sep;88(1-2):163-74. doi: 10.1016/s0166-6851(97)00090-x. PMID: 9274877.

30. MacLennan K, Gallagher MP, Knox DP. Stage-specific serine and metallo-proteinase release by adult and larval *Trichostrongylus vitrinus*. Int J Parasitol. 1997 Sep;27(9):1031-6. doi: 10.1016/s0020-7519(97)00074-x. PMID: 9363485.

31. Chadderdon RC, Cappello M. The hookworm platelet inhibitor: functional blockade of integrins GPIIb/IIIa (alphaIIbbeta3) and GPIa/IIa (alpha2beta1) inhibits platelet aggregation and adhesion *in vitro*. J Infect Dis. 1999 May;179(5):1235-41. doi: 10.1086/314724. PMID: 10191228.

32. Vázquez-López C, de Armas-Serra C, Giménez-Pardo C, Rodríguez-Caabeiro F. Proteolytic activity of the *Gymnorhynchus gigas* plerocercoid: purification and properties of a collagenase from the crude extract. Parasitol Res. 1999 Jan;85(1):64-70. doi: 10.1007/s004360050508. PMID: 9950230.

33. Geldhof P, Claerebout E, Knox DP, Jagneessens J, Vercruysse J. Proteinases released *in vitro* by the parasitic stages of the bovine abomasal nematode *Ostertagia ostertagi*. Parasitology. 2000 Dec;121 Pt 6:639-47. doi: 10.1017/s0031182000006806. PMID: 11155935.

34. Gomez-Escobar N, Gregory WF, Maizels RM. Identification of tgh-2, a filarial nematode homolog of *Caenorhabditis elegans* daf-7 and human transforming growth factor beta, expressed in microfilarial and adult stages of *Brugia malayi*. Infect Immun. 2000 Nov;68(11):6402-10. doi: 10.1128/IAI.68.11.6402-6410.2000. PMID: 11035752; PMCID: PMC97726.

35. Ros-Moreno RM, Vázquez-López C, Giménez-Pardo C, de Armas-Serra C, Rodríguez-Caabeiro F. A study of proteases throughout the life cycle of *Trichinella spiralis*. Folia Parasitol (Praha). 2000;47(1):49-54. doi: 10.14411/fp.2000.009. PMID: 10833016.

36. Buddai SK, Toulokhonova L, Bergum PW, Vlasuk GP, Krishnaswamy S. Nematode anticoagulant protein c2 reveals a site on factor Xa that is important for macromolecular substrate binding to human prothrombinase. J Biol Chem. 2002 Jul 19;277(29):26689-98. doi: 10.1074/jbc.M202507200. Epub 2002 May 14. PMID: 12011050.

37. Crab A, Noppe W, Pelicaen C, Van Hoorelbeke K, Deckmyn H. The parasitic hematophagous worm *Haemonchus contortus* inhibits human platelet aggregation and adhesion: partial purification of a platelet inhibitor. Thromb Haemost. 2002 May;87(5):899-904. PMID: 12038795.

38. Harrison LM, Nerlinger A, Bungiro RD, Córdova JL, Kuzmic P, Cappello M. Molecular characterization of *Ancylostoma* inhibitors of coagulation factor Xa. Hookworm anticoagulant activity in vitro predicts parasite bloodfeeding *in vivo*. J Biol Chem. 2002 Feb 22;277(8):6223-9. doi: 10.1074/jbc.M109908200. Epub 2001 Dec 7. PMID: 11741914.

39. Doenhoff MJ, Stanley RG, Pryce D, Curtis RC, Parry H, Griffiths K, Jackson CL. Identification of a fibrinolytic enzyme in *Schistosoma mansoni* eggs and modulated blood fibrinogen metabolism in *S. mansoni*-infected mice. Parasitology. 2003 Mar;126(Pt 3):231-4. doi: 10.1017/s0031182002002809. PMID: 12666881.

40. Joachim A, Ali SF, Daugschies A. *Fasciola hepatica* alters coagulation parameters in sheep plasma *in vivo* and *in vitro*. Parasitol Res. 2003 Jan;89(1):53-8. doi: 10.1007/s00436-002-0723-3. Epub 2002 Aug 29. PMID: 12474044.

41. Jolodar A, Fischer P, Bergmann S, Büttner DW, Hammerschmidt S, Brattig NW. Molecular cloning of an alpha-enolase from the human filarial parasite *Onchocerca volvulus* that binds human plasminogen. Biochim Biophys Acta. 2003 Jun 19;1627(2-3):111-20. doi: 10.1016/s0167-4781(03)00083-6. PMID: 12818429.

42. Williamson AL, Brindley PJ, Loukas A. Hookworm cathepsin D aspartic proteases: contributing roles in the host-specific degradation of serum proteins and skin macromolecules. Parasitology. 2003 Feb;126(Pt 2):179-85. doi: 10.1017/s0031182002002706. PMID: 12636356.

43. Bernal D, de la Rubia JE, Carrasco-Abad AM, Toledo R, Mas-Coma S, Marcilla A. Identification of enolase as a plasminogen-binding protein in excretory-secretory products of *Fasciola hepatica*. FEBS Lett. 2004 Apr 9;563(1-3):203-6. doi: 10.1016/S0014-5793(04)00306-0. PMID: 15063749.

44. Mieszczanek J, Harrison LM, Cappello M. *Ancylostoma ceylanicum* anticoagulant peptide-1: role of the predicted reactive site amino acid in mediating inhibition of coagulation factors Xa and VIIa. Mol Biochem Parasitol. 2004 Sep;137(1):151-9. doi: 10.1016/j.molbiopara.2004.05.011. PMID: 15279961.

45. Mieszczanek J, Harrison LM, Vlasuk GP, Cappello M. Anticoagulant peptides from *Ancylostoma caninum* are immunologically distinct and localize to separate structures within the adult hookworm. Mol Biochem Parasitol. 2004 Feb;133(2):319-23. doi: 10.1016/j.molbiopara.2003.10.015. PMID: 14698444.

46. Erttmann KD, Kleensang A, Schneider E, Hammerschmidt S, Büttner DW, Gallin M. Cloning, characterization and DNA immunization of an *Onchocerca volvulus* glyceraldehyde-3-phosphate dehydrogenase (Ov-GAPDH). Biochim Biophys Acta. 2005 Jun 30;1741(1-2):85-94. doi: 10.1016/j.bbadis.2004.12.010. Epub 2005 Jan 5. PMID: 15955451.

47. Suchitra S, Joshi P. Characterization of *Haemonchus contortus* calreticulina suggests its role in feeding and immune evasion by the parasite. Biochim Biophys Acta. 2005 Apr 15;1722(3):293-303. doi: 10.1016/j.bbagen.2004.12.020. Epub 2005 Jan 18. PMID: 15716049.

48. Barbosa AP, Campos DM, Semerene AR, Teixeira AR, Santana JM. *Lagochilascaris minor* third-stage larvae secrete metalloproteases with specificity for fibrinogen and native collagen. Microbes Infect. 2006 Oct;8(12-13):2725-32. doi: 10.1016/j.micinf.2006.08.001. Epub 2006 Aug 30. PMID: 16979366.

49. Lin YL, He S. Sm22.6 antigen is an inhibitor to human thrombin. Mol Biochem Parasitol. 2006 May;147(1):95-100. doi: 10.1016/j.molbiopara.2006.01.012. Epub 2006 Feb 14. PMID: 16499980.

50. Muleke CI, Ruofeng Y, Lixin X, Yanming S, Xiangrui L. Characterization of HC58cDNA, a putative cysteine protease from the parasite *Haemonchus contortus*. J Vet Sci. 2006 Sep;7(3):249-55. doi: 10.4142/jvs.2006.7.3.249. Erratum in: J Vet Sci. 2006 Dec;7(4):405. PMID: 16871019; PMCID: PMC3242124.

51. Wang K, Guo Y, Li K, Lu Y, Zhang Y, Sun S, Yan H, Zhang S. Molecular characterization and anticoagulant activity of a novel annexin derived from the *Taenia solium*. Acta Trop. 2006 Oct;99(2-3):165-72. doi: 10.1016/j.actatropica.2006.07.006. Epub 2006 Sep 18. PMID: 16982028.

52. Marcilla A, Pérez-García A, Espert A, Bernal D, Muñoz-Antolí C, Esteban JG, Toledo R. *Echinostoma caproni*: identification of enolase in excretory/secretory products, molecular cloning, and functional expression. Exp Parasitol. 2007 Sep;117(1):57-64. doi: 10.1016/j.exppara.2007.03.011. Epub 2007 Mar 27. PMID: 17462631.

53. Merckelbach A, Ruppel A. Biochemical properties of an intracellular serpin from *Echinococcus multilocularis*. Mol Biochem Parasitol. 2007 Nov;156(1):84-8 doi: 10.1016/j.molbiopara.2007.07.013. Epub 2007 Jul 24. PMID: 17727977.

54. Ramajo-Hernández A, Pérez-Sánchez R, Ramajo-Martín V, Oleaga A. *Schistosoma bovis*: plasminogen binding in adults and the identification of plasminogen-binding proteins from the worm tegument. Exp Parasitol. 2007 Jan;115(1):83-91. doi: 10.1016/j.exppara.2006.07.003. Epub 2006 Sep 8. PMID: 16962583.

55. Wu YP, Lenting PJ, Tielens AG, de Groot PG, van Hellemond JJ. Differential platelet adhesion to distinct life-cycle stages of the parasitic helminth *Schistosoma mansoni*. J Thromb Haemost. 2007 Oct;5(10):2146-8. doi: 10.1111/j.1538-7836.2007.02725.x. PMID: 17883706.

56. Zhang Y, Wang KH, Guo YJ, Lu YM, Yan HL, Song YL, Wang F, Ding FX, Sun SH. Annexin B1 from *Taenia solium* metacestodes is a newly characterized member of the annexin family. Biol Chem. 2007 Jun;388(6):601-10. doi: 10.1515/BC.2007.071. PMID: 17552907.

57. Geldhof P, Knox D. The intestinal contortin structure in *Haemonchus contortus*: an immobilised anticoagulant? Int J Parasitol. 2008 Nov;38(13):1579-88. doi: 10.1016/j.ijpara.2008.05.002. Epub 2008 May 21. PMID: 18599060.

58. Gan W, Deng L, Yang C, He Q, Hu J, Yin H, Jin X, Lu C, Wu Y, Peng L. An anticoagulant peptide from the human hookworm, *Ancylostoma duodenale* that inhibits coagulation factors Xa and XIa. FEBS Lett. 2009 Jun 18;583(12):1976-80. doi: 10.1016/j.febslet.2009.05.009. Epub 2009 May 14. PMID: 19446556.

59. Hwang JH, Lee WG, Na BK, Lee HW, Cho SH, Kim TS. Identification and characterization of a serine protease inhibitor of *Paragonimus westermani*. Parasitol Res. 2009 Feb;104(3):495-501. doi: 10.1007/s00436-008-1219-6. Epub 2008 Oct 17. PMID: 18925417.

60. de la Torre-Escudero E, Manzano-Román R, Pérez-Sánchez R, Siles-Lucas M, Oleaga A. Cloning and characterization of a plasminogen-binding surface-associated enolase from *Schistosoma bovis*. Vet Parasitol. 2010 Oct 11;173(1-2):76-84. doi: 10.1016/j.vetpar.2010.06.011. Epub 2010 Jun 15. PMID: 20609522.

61. Deng L, He Q, Kang T, Yin H, Jin X, Li H, Gan W, Yang C, Hu J, Wu Y, Peng L. Identification of an anticoagulant peptide that inhibits both fXIa and fVIIa/tissue factor from the blood-feeding nematode *Ancylostoma caninum*. Biochem Biophys Res Commun. 2010 Feb 5;392(2):155-9. doi: 10.1016/j.bbrc.2009.12.177. Epub 2010 Jan 7. PMID: 20059979.

62. Lu YM, Wang N, Wang JJ, Wang KH, Sun SH. Expression, purification, and characterization of a novel Ca(2+)- and phospholipid-binding protein annexin B2. Mol Biol Rep. 2010 Mar;37(3):1591-6. doi: 10.1007/s11033-009-9568-5. Epub 2009 May 20. PMID: 19455404; PMCID: PMC2815297.

63. Yang J, Qiu C, Xia Y, Yao L, Fu Z, Yuan C, Feng X, Lin J. Molecular cloning and functional characterization of *Schistosoma japonicum* enolase which is highly expressed at the schistosomulum stage. Parasitol Res. 2010 Aug;107(3):667-77. doi: 10.1007/s00436-010-1913-z. Epub 2010 May 29. PMID: 20512506.

64. Yi D, Xu L, Yan R, Li X. *Haemonchus contortus*: cloning and characterization of serpin. Exp Parasitol. 2010 Aug;125(4):363-70. doi: 10.1016/j.exppara.2010.03.002. Epub 2010 Mar 7. PMID: 20214897.

65. Jiang D, Zhan B, Mayor RS, Gillespie P, Keegan B, Bottazzi ME, Hotez P. Ac-AP-12, a novel factor Xa anticoagulant peptide from the esophageal glands of adult *Ancylostoma caninum*. Mol Biochem Parasitol. 2011 May;177(1):42-8. doi: 10.1016/j.molbiopara.2011.01.008. Epub 2011 Jan 18. PMID: 21251931.

66. Wang X, Chen W, Hu F, Deng C, Zhou C, Lv X, Fan Y, Men J, Huang Y, Sun J, Hu D, Chen J, Yang Y, Liang C, Zheng H, Hu X, Xu J, Wu Z, Yu X. *Clonorchis sinensi*s enolase: identification and biochemical characterization of a glycolytic enzyme from excretory/secretory products. Mol Biochem Parasitol. 2011 Jun;177(2):135-42. doi: 10.1016/j.molbiopara.2011.02.011. Epub 2011 Mar 4. PMID: 21382423.

67. de la Torre-Escudero E, Manzano-Román R, Siles-Lucas M, Pérez-Sánchez R, Moyano JC, Barrera I, Oleaga A. Molecular and functional characterization of a *Schistosoma bovis* annexin: fibrinolytic and anticoagulant activity. Vet Parasitol. 2012 Feb 28;184(1):25-36. doi: 10.1016/j.vetpar.2011.08.013. Epub 2011 Aug 16. PMID: 21889851.

68. González-Miguel J, Morchón R, Mellado I, Carretón E, Montoya-Alonso JA, Simón F. Excretory/secretory antigens from *Dirofilaria immitis* adult worms interact with the host fibrinolytic system involving the vascular endothelium. Mol Biochem Parasitol. 2012 Feb;181(2):134-40. doi: 10.1016/j.molbiopara.2011.10.010. Epub 2011 Oct 25. PMID: 22050927.

69. Tong Y, Zhou J, Mao M, Gao J, Yuan L. Generation of bioactive recombinant *Ancylostoma caninum* anticoagulant peptide c2. Protein Expr Purif. 2012 Jan;81(1):49-54. doi: 10.1016/j.pep.2011.08.029. Epub 2011 Aug 31. PMID: 21907287.

70. González-Miguel J, Morchón R, Carretón E, Montoya-Alonso JA, Simón F. Surface associated antigens of *Dirofilaria immitis* adult worms activate the host fibrinolytic system. Vet Parasitol. 2013 Sep 1;196(1-2):235-40. doi: 10.1016/j.vetpar.2013.01.028. Epub 2013 Feb 4. PMID: 23433649.

71. Lei H, Tian Y, Chen W, Wang X, Li X, Mao Q, Sun J, Li R, Xu Y, Liang C, Huang Y, Yu X. The biochemical and immunological characterization of two serpins from *Clonorchis sinensis*. Mol Biol Rep. 2013 Jun;40(6):3977-85. doi: 10.1007/s11033-012-2475-1. Epub 2012 Dec 30. PMID: 23275238.

72. deWalick S, Hensbergen PJ, Bexkens ML, Grosserichter-Wagener C, Hokke CH, Deelder AM, de Groot PG, Tielens AG, van Hellemond JJ. Binding of von Willebrand factor and plasma proteins to the eggshell of *Schistosoma mansoni*. Int J Parasitol. 2014 Apr;44(5):263-8. doi: 10.1016/j.ijpara.2013.12.006. Epub 2014 Feb 18. PMID: 24560918.

73. He L, Ren M, Chen X, Wang X, Li S, Lin J, Liang C, Liang P, Hu Y, Lei H, Bian M, Huang Y, Wu Z, Li X, Yu X. Biochemical and immunological characterization of annexin B30 from *Clonorchis sinensis* excretory/secretory products. Parasitol Res. 2014 Jul;113(7):2743-55. doi: 10.1007/s00436-014-3935-4. Epub 2014 May 27. PMID: 24861011.

74. Hu Y, Zhang E, Huang L, Li W, Liang P, Wang X, Xu J, Huang Y, Yu X. Expression profiles of glyceraldehyde-3-phosphate dehydrogenase from *Clonorchis sinensis*: a glycolytic enzyme with plasminogen binding capacity. Parasitol Res. 2014 Dec;113(12):4543-53. doi: 10.1007/s00436-014-4144-x. Epub 2014 Oct 10. PMID: 25300416.

75. Figueiredo BC, Da'dara AA, Oliveira SC, Skelly PJ. Schistosomes Enhance Plasminogen Activation: The Role of Tegumental Enolase. PLoS Pathog. 2015 Dec 11;11(12):e1005335. doi: 10.1371/journal.ppat.1005335. PMID: 26658895; PMCID: PMC4676649.

76. González-Miguel J, Morchón R, Carretón E, Montoya-Alonso JA, Simón F. Can the activation of plasminogen/plasmin system of the host by metabolic products of *Dirofilaria immitis* participate in heartworm disease endarteritis? Parasit Vectors. 2015 Apr 1;8:194. doi: 10.1186/s13071-015-0799-0. PMID: 25888952; PMCID: PMC4391138.

77. González-Miguel J, Morchón R, Siles-Lucas M, Oleaga A, Simón F. Surface- displayed glyceraldehyde 3-phosphate dehydrogenase and galectin from *Dirofilaria immitis* enhance the activation of the fibrinolytic system of the host. Acta Trop. 2015 May;145:8-16. doi: 10.1016/j.actatropica.2015.01.010. Epub 2015 Feb 7. PMID: 25666684.

78. González-Miguel J, Morchón R, Siles-Lucas M, Simón F. Fibrinolysis and proliferative endarteritis: two related processes in chronic infections? The model of the blood-borne pathogen *Dirofilaria immitis*. PLoS One. 2015 Apr 13;10(4):e0124445. doi: 10.1371/journal.pone.0124445. PMID: 25875022; PMCID: PMC4395379.

79. Li WH, Qu ZG, Zhang NZ, Yue L, Jia WZ, Luo JX, Yin H, Fu BQ. Molecular characterization of enolase gene from *Taenia multiceps*. Res Vet Sci. 2015 Oct;102:53-8. doi: 10.1016/j.rvsc.2015.06.013. Epub 2015 Jul 2. PMID: 26412520.

80. Ranasinghe SL, Fischer K, Gobert GN, McManus DP. A novel coagulation inhibitor from *Schistosoma japonicum*. Parasitology. 2015 Dec;142(14):1663-72. doi: 10.1017/S0031182015001328. Epub 2015 Oct 14. PMID: 26463744.

81. Ranasinghe SL, Fischer K, Gobert GN, McManus DP. Functional expression of a novel Kunitz type protease inhibitor from the human blood fluke *Schistosoma mansoni*. Parasit Vectors. 2015 Aug 4;8:408. doi: 10.1186/s13071-015-1022-z. PMID: 26238343; PMCID: PMC4524284.

82. Tong Y, Yue J, Mao M, Liu Q, Zhou J, Yang J. Recombinant nematode anticoagulant protein c2 inhibits cell invasion by decreasing uPA expression in NSCLC cells. Oncol Rep. 2015 Apr;33(4):1815-22. doi: 10.3892/or.2015.3795. Epub 2015 Feb 10. PMID: 25672417.

83. Valdivieso E, Perteguer MJ, Hurtado C, Campioli P, Rodríguez E, Saborido A, Martínez-Sernández V, Gómez-Puertas P, Ubeira FM, Gárate T. ANISERP: a new serpin from the parasite *Anisakis simplex*. Parasit Vectors. 2015 Jul 28;8:399 doi: 10.1186/s13071-015-1006-z. PMID: 26215984; PMCID: PMC4517634.

84. Zhang S, Guo A, Zhu X, You Y, Hou J, Wang Q, Luo X, Cai X. Identification and functional characterization of alpha-enolase from *Taenia pisiformis* metacestode. Acta Trop. 2015 Apr;144:31-40. doi: 10.1016/j.actatropica.2015.01.007. Epub 2015 Jan 23. PMID: 25623259.

85. Zhu Y, Lin Y, Liu A, Shui M, Li R, Liu X, Hu W, Wang Y. Structure-guided creation of AcAP5-derived and platelet targeted factor Xa inhibitors. Biochem Pharmacol. 2015 Jun 15;95(4):253-62. doi: 10.1016/j.bcp.2015.04.004. Epub 2015 Apr 15. PMID: 25887920.

86. Da'dara AA, de Laforcade AM, Skelly PJ. The impact of schistosomes and schistosomiasis on murine blood coagulation and fibrinolysis as determined by thromboelastography (TEG). J Thromb Thrombolysis. 2016 May;41(4):671-7. doi: 10.1007/s11239-015-1298-z. PMID: 26573180; PMCID: PMC5467217.

87. Takashima Y, Onoda I, Chiou SP, Kitoh K. In vitro canine platelet aggregation caused by *Dirofilaria immitis* extract. J Vet Med Sci. 2017 Feb 28;79(2):387-392. doi: 10.1292/jvms.16-0461. Epub 2016 Dec 30. PMID: 28049921; PMCID: PMC5326946.

88. Ayón-Núñez DA, Fragoso G, Espitia C, García-Varela M, Soberón X, Rosas G, Laclette JP, Bobes RJ. Identification and characterization of *Taenia solium* enolase as a plasminogen-binding protein. Acta Trop. 2018 Jun;182:69-79. doi: 10.1016/j.actatropica.2018.02.020. Epub 2018 Feb 18. PMID: 29466706.

89. Elzoheiry M, Da'dara AA, deLaforcade AM, El-Beshbishi SN, Skelly PJ. The Essential Ectoenzyme SmNPP5 from the Human Intravascular Parasite *Schistosoma mansoni* is an ADPase and a Potent Inhibitor of Platelet Aggregation. Thromb Haemost. 2018 Jun;118(6):979-989. doi: 10.1055/s-0038-1641715. Epub 2018 Apr 18. PMID: 29669386.

90. Fernandes RS, Fernandes LGV, de Godoy AS, Miyasato PA, Nakano E, Farias LP, Nascimento ALTO, Leite LCC. *Schistosoma mansoni* venom allergen-like protein 18 (SmVAL18) is a plasminogen-binding protein secreted during the early stages of mammalian-host infection. Mol Biochem Parasitol. 2018 Apr;221:23-31. doi: 10.1016/j.molbiopara.2018.02.003. Epub 2018 Feb 22. PMID: 29477861.

91. Leontovyč A, Ulrychová L, O'Donoghue AJ, Vondrášek J, Marešová L, Hubálek M, Fajtová P, Chanová M, Jiang Z, Craik CS, Caffrey CR, Mareš M, Dvořák J, Horn M. SmSP2: A serine protease secreted by the blood fluke pathogen *Schistosoma mansoni* with anti-hemostatic properties. PLoS Negl Trop Dis. 2018 Apr 20;12(4):e0006446. doi: 10.1371/journal.pntd.0006446. PMID: 29677188; PMCID: PMC5931690.

92. Mebius MM, Op Heij JMJ, Tielens AGM, de Groot PG, Urbanus RT, van Hellemond JJ. Fibrinogen and fibrin are novel substrates for *Fasciola hepatica* cathepsin L peptidases. Mol Biochem Parasitol. 2018 Apr;221:10-13. doi: 10.1016/j.molbiopara.2018.02.001. Epub 2018 Feb 4. PMID: 29414671.

93. Zhang Y, Guo J, He L, Zong HY, Cai GB. Isolation and characterization of a novel serine protease inhibitor, SjSPI, from *Schistosoma japonicum*. Parasitol Int. 2018 Aug;67(4):415-424. doi: 10.1016/j.parint.2018.04.002. Epub 2018 Apr 9. PMID: 29649563.

94. Zhang S, You Y, Luo X, Zheng Y, Cai X. Molecular and biochemical characterization of *Taenia solium* α-enolase. Vet Parasitol. 2018 Apr 30;254:36-42. doi: 10.1016/j.vetpar.2018.02.041. Epub 2018 Feb 25. PMID: 29657009.

95. González-Miguel J, Valero MA, Reguera-Gomez M, Mas-Bargues C, Bargues MD, Simón F, Mas-Coma S. Numerous *Fasciola* plasminogen-binding proteins may underlie blood-brain barrier leakage and explain neurological disorder complexity and heterogeneity in the acute and chronic phases of human fascioliasis. Parasitology. 2019 Mar;146(3):284-298. doi: 10.1017/S0031182018001464. Epub 2018 Sep 24. Erratum in: Parasitology. 2020 May;147(6):729. PMID: 30246668; PMCID: PMC6402360.

96. Jiang P, Zao YJ, Yan SW, Song YY, Yang DM, Dai LY, Liu RD, Zhang X, Wang ZQ, Cui J. Molecular characterization of a *Trichinella spiralis* enolase and its interaction with the host's plasminogen. Vet Res. 2019 Dec 5;50(1):106. doi: 10.1186/s13567-019-0727-y. PMID: 31806006; PMCID: PMC6894503.
