# Supplementary material for: Interaction of helminth parasites with the haemostatic system of their vertebrate hosts: a scoping review
Source: Parasite. 2022 Jul 14;29:35. doi: 10.1051/parasite/2022034 (PMC9281497; doi:10.1051/parasite/2022034)
Supplement: Supplementary file 6 — Supplementary Table 1. Interactions between each helminth parasite species and the host haemostatic system. [file parasite-29-35-s6.pdf]

Supplementary Table 1. Interaction of helminth parasites with the haemostatic system of their vertebrate hosts: a scoping review. Alicia Diosdado, Fernando Simón, Judit Serrat, Javier González-Miguel. Parasite.

Helminth parasite species	Type of interaction
Ancylostoma caninum	• Inhibition of platelet aggregation<br>• Inhibition of the extrinsic, intrinsic and/or common pathways of the coagulation cascade<br>• Inhibition of FVIIa/TF<br>• Inhibition of FXIa<br>• Inhibition of FXa<br>• Inhibition of FXa/FVa<br>• Binding of FX<br>• Binding of FXa<br>• Degradation of FG<br>• Degradation of PLG<br>• Decrease in uPA expression
Ancylostoma ceylanicum	• Inhibition of platelet aggregation<br>• Inhibition of the extrinsic, intrinsic and/or common pathways of the coagulation cascade<br>• Inhibition of FVIIa/TF<br>• Inhibition of FXa<br>• Binding of FXa
Ancylostoma duodenale	• Inhibition of the extrinsic, intrinsic and/or common pathways of the coagulation cascade<br>• Inhibition of FXIa<br>• Inhibition of FXa
Anisakis simplex	• Inhibition of the extrinsic, intrinsic and/or common pathways of the coagulation cascade<br>• Inhibition of thrombin
Ascaris lumbricoides	• Inhibition of the extrinsic, intrinsic and/or common pathways of the coagulation cascade<br>• Inhibition of the conversion of FG into fibrin
Ascaris suum	• Inhibition of platelet aggregation<br>• Inhibition of the intrinsic and/or common pathways of the coagulation cascade
Brugia malayi	• Inhibition of platelet aggregation<br>• Increase in PAI-1 transcription
Clonorchis sinensis	• Inhibition of thrombin<br>• Binding of PLG

Dirofilaria immitis	• Induction of platelet aggregation<br>• Inhibition of platelet aggregation<br>• Inhibition of platelet activation<br>• Degradation of FG<br>• Binding of PLG<br>• Activation of PLG (by tPA)<br>• Increase in tPA expression<br>• Increase in uPA expression<br>• Decrease in PAI-1 expression
Dracunculus medinensis	• Activation of PLG
Echinococcus multilocularis	• Inhibition of plasmin
Echinostoma caproni	• Binding of PLG
Fasciola hepatica	• Inhibition of the extrinsic and/or common pathways of the coagulation cascade<br>• Inhibition of the conversion of FG into fibrin<br>• Activation of the intrinsic and/or common pathways of the coagulation cascade<br>• Degradation of FG<br>• Degradation of fibrin<br>• Binding of PLG<br>• Activation of PLG (by tPA)
Gymnorhynchus gigas	• Degradation of FG
Haemonchus contortus	• Inhibition of platelet aggregation<br>• Inhibition of coagulation<br>• Inhibition of the conversion of FG into fibrin<br>• Activation of the intrinsic and/or common pathways of the coagulation cascade<br>• Binding of FXa<br>• Degradation of FG<br>• Inhibition of fibrin re-aggregation
Lagochilascaris minor	• Degradation of FG
Necator americanus	• Inhibition of platelet aggregation<br>• Inhibition of platelet-granule release<br>• Inhibition of the extrinsic, intrinsic and/or common pathways of the coagulation cascade<br>• Inhibition of FXa<br>• Degradation of FG
Onchocerca cervicalis	• Induction of platelet aggregation
Onchocerca volvulus	• Binding of PLG
Ostertagia ostertagi	• Degradation of FG
Paragonimus westermani	• Inhibition of thrombin
Schistosoma bovis	• Inhibition of the extrinsic, intrinsic and/or common pathways of the coagulation cascade<br>• Binding of PLG<br>• Activation of PLG (by tPA and without PLG activator)

Schistosoma haematobium	• Activation of PLG (by tPA)
Schistosoma japonicum	• Inhibition of the intrinsic and/or common pathways of the coagulation cascade<br>• Inhibition of FXa<br>• Inhibition of thrombin<br>• Binding of PLG<br>• Activation of PLG (by tPA)
Schistosoma mansoni	• Binding of vWF<br>• Induction of platelet adhesion<br>• Inhibition of platelet adhesion<br>• Inhibition of platelet aggregation<br>• Inhibition of the extrinsic, intrinsic and/or common pathways of the coagulation cascade<br>• Inhibition of FXIIa<br>• Inhibition of FXa<br>• Inhibition of thrombin<br>• Binding of thrombin<br>• Binding of FG<br>• Degradation of FG<br>• Degradation of fibrin<br>• Binding of PLG<br>• Activation of PLG (by tPA and uPA)<br>• Activation of tPA
Taenia multiceps	• Binding of PLG
Taenia pisiformis	• Binding of PLG<br>• Activation of PLG (by uPA)
Taenia solium	• Binding of platelets<br>• Inhibition of platelet aggregation<br>• Inhibition of the extrinsic, intrinsic and/or common pathways of the coagulation cascade<br>• Binding of PLG<br>• Activation of PLG (by tPA)
Taenia taeniaeformis	• Inhibition of the intrinsic and/or common pathways of the coagulation cascade
Teladorsagia circumcincta	• Degradation of FG<br>• Degradation of PLG
Trichinella spiralis	• Degradation of FG<br>• Binding of PLG<br>• Degradation of PLG
Trichostrongylus vitrinus	• Degradation of FG<br>• Degradation of PLG

Interactions with the coagulation system and the fibrinolytic system are coloured orange and blue, respectively.

Abbreviations: FVIIa/TF: activated coagulation factor VII/tissue factor complex; FXIa: activated coagulation factor XI; FXa: activated coagulation factor X; FXa/FVa: activated coagulation factor X/activated coagulation factor V complex; FX: coagulation factor X; FG: fibrinogen; PLG: plasminogen; uPA: urokinase-type plasminogen activator; PAI-1: plasminogen activator inhibitor 1; tPA: tissue plasminogen activator; vWF: von Willebrand Factor; FXIIa: activated coagulation factor XII.
