# Supplementary material for: Interaction of helminth parasites with the haemostatic system of their vertebrate hosts: a scoping review
Source: Parasite. 2022 Jul 14;29:35. doi: 10.1051/parasite/2022034 (PMC9281497; doi:10.1051/parasite/2022034)
Supplement: Supplementary file 7 — Supplementary Table 2. Helminth species, stage and protein compartment in which each interacting parasite molecule was identified and type of interaction with the haemostatic system. [file parasite-29-35-s7.pdf]

Supplementary Table 2. Interaction of helminth parasites with the haemostatic system of their vertebrate hosts: a scoping review. Alicia Diosdado, Fernando Simón, Judit Serrat, Javier González-Miguel. Parasite.

**Parasite molecule**	**Helminth parasite species**	**Stage**	**Protein compartment**	**Type of interaction**
Actin	*D. immitis*<br>*S. bovis*<br>*T. solium*	A, L	So, S, ES	• Binding of PLG<br>• Activation of PLG (by tPA)<br>• Increase in tPA expression<br>• Increase in uPA expression
Adenylate kinase	*S. bovis*	A	S	• Binding of PLG
Ancylostoma spp. anticoagulant peptide/protein	*A. caninum*<br>*A. ceylanicum*<br>*A. duodenale*	A	So	• Inhibition of platelet aggregation<br>• Inhibition of the extrinsic, intrinsic and/or common pathways of the coagulation cascade<br>• Inhibition of FVIIa/TF<br>• Inhibition of FXIa<br>• Inhibition of FXa<br>• Inhibition of FXa/FVa<br>• Binding of FX<br>• Binding of FXa<br>• Decrease in uPA expression
Annexin	*C. sinensis*<br>*S. bovis*<br>*T. solium*	A, L	So	• Binding of platelets<br>• Inhibition of platelet aggregation<br>• Inhibition of the extrinsic, intrinsic and/or common pathways of the coagulation cascade<br>• Binding of PLG<br>• Activation of PLG (by tPA)
Aspartic protease	*H. contortus*<br>*N. americanus*<br>*O. ostertagi*<br>*T. circumcincta*<br>*T. spiralis*	A, L	So, ES	• Degradation of FG
ATP:guanidino kinase	*S. bovis*	A	S	• Binding of PLG
Beta-galactosidase-binding-lectin	*D. immitis*	A	S	• Binding of PLG
Calpain	*F. hepatica*	A	ES	• Inhibition of the extrinsic and/or common pathways of the coagulation cascade<br>• Inhibition of the conversion of FG into fibrin<br>• Activation of the intrinsic and/or common pathways of the coagulation cascade
Calreticulin	*H. contortus*	A	So	• Inhibition of coagulation<br>• Binding of FXa

Carbohydrates	*D. immitis*	A	–	• Inhibition of platelet aggregation<br>• Inhibition of platelet activation
Cathepsin	*A. caninum*<br>*F. hepatica*<br>*N. americanus*	A	ES	• Degradation of FG<br>• Degradation of fibrin<br>• Binding of PLG
Chaperonin protein HSP60	*D. immitis*	A	ES	• Binding of PLG
Collagen	*O. cervicalis*	A	S	• Induction of platelet aggregation
Cyclophilin	*D. immitis*	A	S	• Binding of PLG
Cysteine protease	*F. hepatica*<br>*G. gigas*<br>*H. contortus*<br>*O. ostertagi*<br>*T. spiralis*	A, L	So, ES	• Inhibition of the extrinsic and/or common pathways of the coagulation cascade<br>• Inhibition of the conversion of FG into fibrin<br>• Activation of the intrinsic and/or common pathways of the coagulation cascade<br>• Degradation of FG
Cytosolic malate dehydrogenase	*T. solium*	L	So	• Binding of PLG
Dihydrolipoyl dehydrogenase	*F. hepatica*	A	ES	• Binding of PLG
Dipeptidyl peptidase IV	*H. contortus*	A	So	• Degradation of FG<br>• Inhibition of fibrin re-aggregation
Ectonucleotide pyrophosphatase/phosphodiesterase	*S. mansoni*	A	–	• Inhibition of platelet aggregation
Enolase	*C. sinensis*<br>*D. immitis*<br>*E. caproni*<br>*F. hepatica*<br>*O. volvulus*<br>*S. bovis*<br>*S. japonicum*<br>*S. mansoni*<br>*T. multiceps*<br>*T. pisiformis*<br>*T. solium*<br>*T. spiralis*	A, L	So, S, ES	• Binding of PLG<br>• Activation of PLG (by tPA and uPA)
Fasciclin	*T. solium*	L	So	• Binding of PLG
Fatty acid-binding protein	*F. hepatica*	A	ES	• Binding of PLG
Fructose-bisphosphate aldolase	*D. immitis*<br>*F. hepatica*<br>*S. bovis*	A	S, ES	• Binding of PLG<br>• Activation of PLG (by tPA)<br>• Increase in tPA expression<br>• Increase in uPA expression

Galectin	*D. immitis*	A	S, ES	• Binding of PLG<br>• Activation of PLG (by tPA)<br>• Increase in uPA expression
Glutamate dehydrogenase	*F. hepatica*	A	ES	• Binding of PLG
Glutathione S-transferase	*F. hepatica*	A	ES	• Binding of PLG
Glyceraldehyde-3-phosphate dehydrogenase	*C. sinensis*<br>*D. immitis*<br>*F. hepatica*<br>*O. volvulus*<br>*S. bovis*	A	S, ES	• Binding of PLG<br>• Activation of PLG (by tPA)<br>• Increase in uPA expression
Hookworm platelet inhibitor	*A. caninum*	A	So	• Inhibition of platelet aggregation
Hypothetical (Clone ZZD114 mRNA sequence)	*S. bovis*	A	S	• Binding of PLG
Hypothetical protein	*S. bovis*	A	S	• Binding of PLG
Hypothetical protein LOAG_14743	*D. immitis*	A	ES	• Binding of PLG
Hypothetical protein T265_01567	*F. hepatica*	A	ES	• Binding of PLG
Immunoglobulin I-set domain-containing protein	*D. immitis*	A	S	• Binding of PLG
Kunitz protein	*S. japonicum*<br>*S. mansoni*	A	–	• Inhibition of the extrinsic, intrinsic and/or common pathways of the coagulation cascade<br>• Inhibition of FXa
Major sperm protein	*D. immitis*	A	S	• Binding of PLG
Metalloprotease	*F. hepatica*<br>*H. contortus*<br>*O. ostertagi*<br>*T. circumcincta*<br>*T. vitrinus*	A, L	ES	• Inhibition of the extrinsic and/or common pathways of the coagulation cascade<br>• Inhibition of the conversion of FG into fibrin<br>• Activation of the intrinsic and/or common pathways of the coagulation cascade<br>• Degradation of FG<br>• Degradation of PLG
Mitogen activated protein kinase	*T. solium*	L	So	• Binding of PLG
MSP domain protein with Glu-rich domain	*D. immitis*	A	S	• Binding of PLG
Ov87	*D. immitis*	A	ES	• Binding of PLG
P22U	*D. immitis*	A	ES	• Binding of PLG
Phosphoglycerate mutase	*S. bovis*	A	S	• Binding of PLG
Plasmin-like enzyme	*S. mansoni*	E	So	• Degradation of FG
Procathepsin L1	*F. hepatica*	A	ES	• Binding of PLG
Protein disulphide isomerase	*F. hepatica*	A	ES	• Binding of PLG

Serine protease	*F. hepatica*<br>*G. gigas*<br>*H. contortus*<br>*N. americanus*<br>*O. ostertagi*<br>*S. mansoni*<br>*T. spiralis*	A, E, L	So, ES	• Inhibition of the extrinsic and/or common pathways of the coagulation cascade<br>• Inhibition of the conversion of FG into fibrin<br>• Activation of the intrinsic and/or common pathways of the coagulation cascade<br>• Degradation of FG<br>• Degradation of fibrin<br>• Activation of PLG<br>• Degradation of PLG<br>• Activation of tPA
Serpin	*A. simplex*<br>*C. sinensis*<br>*E. multilocularis*<br>*H. contortus*<br>*P. westermani*<br>*S. japonicum*	A, L	So	• Inhibition of coagulation<br>• Inhibition of thrombin<br>• Inhibition of plasmin
SJCHGC01945 protein	*F. hepatica*	A	ES	• Binding of PLG
Sm22.6 antigen	*S. mansoni*	–	–	• Inhibition of coagulation<br>• Binding of thrombin<br>• Inhibition of thrombin
Thiol-specific antioxidant protein	*F. hepatica*	A	ES	• Binding of PLG
Thioredoxin-glutathione reductase	*F. hepatica*	A	ES	• Binding of PLG
Transforming growth factor b homolog	*B. malayi*	L	–	• Activation of PAI-1 transcription
Transglutaminase precursor	*D. immitis*	A	ES	• Binding of PLG
Triose phosphate isomerase	*F. hepatica*<br>*S. bovis*	A	S, ES	• Binding of PLG
Venom allergen-like protein	*S. mansoni*	–	ES	• Binding of thrombin<br>• Binding of PLG<br>• Activation of PLG (by uPA)

Interactions with the coagulation system and the fibrinolytic system are coloured orange and blue, respectively.

Abbreviations: A: adult; L: larva; E: egg; So: somatic; S: surface; ES: excretory/secretory; PLG: plasminogen; tPA: tissue plasminogen activator; uPA: urokinase-type plasminogen activator; FVIIa/TF: activated coagulation factor VII/tissue factor complex; FXIa: activated coagulation factor XI; FXa: activated coagulation factor X; FXa/FVa: activated coagulation factor X/activated coagulation factor V complex; FX: coagulation factor X; FG: fibrinogen; PAI-1: plasminogen activator inhibitor 1.
